# Prevalence, Risk Factors, and Genotypes of *Toxoplasma gondii* in Food Animals and Humans (2000–2017) From China

**DOI:** 10.3389/fmicb.2018.02108

**Published:** 2018-09-11

**Authors:** Hui Dong, Ruijing Su, Yaoyao Lu, Mengyao Wang, Jing Liu, Fuchun Jian, Yurong Yang

**Affiliations:** ^1^College of Animal Science and Veterinary Medicine, Henan Agricultural University, Zhengzhou, China; ^2^College of Veterinary Medicine, China Agricultural University, Beijing, China

**Keywords:** *Toxoplasma gondii*, sheep, goats, swines, chickens, cattle, humans, China

## Abstract

*Toxoplasma gondii* as a food-borne pathogen, the infection of it in food animals has relation with human toxoplasmosis, but the trends and epidemiological features of *T. gondii* infections in food animals are rarely studied in China. The aimed of this study was to assess the epidemiology and risks of *T. gondii* in sheep, goats, swines, chickens, yaks, cattle and humans from 2000 to 2017 and to explore prevention and control strategies. The overall seroprevalence of *T. gondii* infections in food animals is 23.7% (39,194/165,417, 95%CI, 23.49–23.90%), which is significantly higher than that in humans (8.2%, 95%CI, 8.06–8.39%, 8,502/103,383) (*P* < 0.0001). Compared the prevalence of *T. gondii* infections in animals and humans sampled from 2000 to 2010, it was significantly increased in the period 2011 to 2017 (*P* < 0.0001). Compared the food animals from non-Yangtze River, animals from regions of the Yangtze River have high seroprevalence rates for *T. gondii* (*P* < 0.0001). Furthermore, samples from the western to eastern regions of the Yellow River showed an increase in seroprevalence for *T. gondii* (*P* < 0.0001). It was speculated that *T. gondii* oocysts may be transmitted by water and annual precipitation possible help the oocyst spread and retain accessible for potential hosts. Effective prevention and control strategies are including water filtration or water boiling, inactivating oocysts from feline’s feces, monitoring birds and rodents. *Chinese 1* (ToxoDB#9) is the predominant genotype in food animals from China.

## Background

Toxoplasmosis, which is caused by *Toxoplasma gondii*, is one of the most common zoonoses around the world, affecting warm-blooded animals, including humans (Zhou et al., 2008; Dubey, 2010). It is estimated that about one-third of the world’s population has been infected with *T. gondii* (Montoya and Liesenfeld, 2004; Weiss and Dubey, 2009). In general, *T. gondii* infections are subclinical or asymptomatic in immunocompetent individuals (Wang et al., 2017). However, *T. gondii* infections involving pregnant women and small ruminants induce abortion or fetal developmental disorders (Dubey, 2010). *T. gondii* also poses a high risk in immunocompromised individuals for severe health problems (Weiss and Dubey, 2009; Wang et al., 2017).

Oocysts, bradyzoites, and tachyzoites are the three infectious stages of *T. gondii*. The transmission route of *T. gondii* may be vertical and horizontal. Vertical transmission involves tachyzoite infection through the placenta or semen (de Moraes et al., 2010; Dubey, 2010; Lopes et al., 2013). Horizontal transmission involves ingestion of fruits, vegetables, or water contaminated with oocysts, tachyzoite infection by blood transfusion or ingestion of raw milk and cheese (Dubey et al., 2014), or consumption of undercooked meat containing bradyzoites. The China annual production of pork, chicken, mutton, and beef has been estimated at 55, 18, 4 and 7 million tones, respectively (National Bureau of Statistics of China, 2015 update). Meats have become the major and favored food in the past decades (Jiang et al., 2013; Wang et al., 2015, 2016; Yang et al., 2017b). Simmered meat, rice, noodles, and mass-cooked vegetables are the main ingredients in the traditional Chinese diet. Raw or undercooked meat is not common in the daily diets of most Chinese. However, undercooked animal products such as hotpot, barbecue, and raw milk are popular dishes in certain regions (Li et al., 2009; Gao and Tang, 2018).

Raw or undercooked meat consumption is significantly associated with human *T. gondii* infections (Kapperud et al., 1996; Tao et al., 2011; Belluco et al., 2017). This is dependent on parasite prevalence, dietary habits, and cultural habits. However, no meat inspection strategy for *T. gondii* contamination has been established, and no performance standards for processing *T. gondii*-positive meat have been developed to date. National surveys have found that the seroprevalence of *T. gondii* infection in humans was increased from 5.2% (1988–1992) to 7.9% (2001–2004) (Yu et al., 1994; Zhou et al., 2008, 2011). The prevalence of *T. gondii* in pregnant Chinese women is less than 10%, indicating that a high proportion of women are susceptible to infection during pregnancy. Approximately 0.3% of pregnant women have been diagnosed with an acute infected of *T. gondii* during pregnancy (1990–2010) (Gao et al., 2012). Pan et al. (2017) have shown that the prevalence of animal *T. gondii* infections in sheep, swines, and chickens in China from 2010 to 2017 is significantly higher than that in humans. Most reports about *T. gondii* from China have been published in local journals, which are generally inaccessible to readers around the world. Therefore, this review aimed to collect all the data about food animals (sheep, goats, swines, chickens, cattle) and humans with *T. gondii* infection, mainly focusing on epidemiological data such as seroprevalence, risk factors, and genotypes in order to provide suggestions on the prevention and control of this foodborne pathogen.

## Data Collection and Statistical Analysis

All the data about food animals and human with *T. gondii* were collected (2000–2017). The references were obtained from Baidu Scholar, China National Knowledge Infrastructure (CNKI), Wan Fang, VIP Chinese Journal Database, PubMed, and Google Scholar. The literature includes criteria were published in English and Chinese, the seroprevalence of *T. gondii* reports was limited to the detection of specific anti-*Toxoplasma* immunoglobulin (Ig) G in serum. The criteria used to exclude the reviewed studies and duplicated reports in the same research group, incomplete original data and the reports published before 2000.

Serological detection of specific anti-*Toxoplasma* immunoglobulin (Ig) G in serum is currently the most widely used method of detecting *T. gondii* in China, which include indirect hemagglutination (IHA), enzyme-linked immunoabsorbent assay (ELISA), modified agglutination test (MAT), latex agglutination test (LAT), and test paper. However, there were few studies compared the sensitivity and specificity of serologic assays for *T. gondii* in different hosts and serological detection methods. Apparent seroprevalence was used to estimate the epidemiological regularity of *T. gondii* infection. The results only represent rough estimates of *T. gondii* infection in China.

To further show epidemiological regularity with geographical differences in China, the country was divided into seven geographical areas. Statistical analysis was performed by the GraphPad Prism 4.0 software (GraphPad Software Inc., San Diego, CA, United States). The data were analyzed by the Chi-square test to determine the association between *T. gondii* seroprevalence with geographic locations and years. *P* < 0.05 was considered statistically significant. Graphs were generated using Graph Pad Prism 7.0 software (GraphPad Software, Inc., San Diego, CA, United States).

## *T. gondii* Prevalence and Risk Assessment in Food Animals and Humans

Felids were the definitive host of *T. gondii*, which spread oocysts by fecal excretion (Dubey, 2010; Dubey et al., 2012). Ding reviewed the published articles from 1995 to 2016 and reported that the seroprevalence in cats was 24.5%, which involved 7,285 cats from 15 provinces in China (Ding et al., 2017). This finding indicates that *T. gondii* oocysts are widely distributed in the country. This viewpoint was also verified by other reports (Pan et al., 2017; Yang et al., 2017a). Furthermore, the popularity of hotpot with undercooked mutton and pork increases the risk of human *T. gondii* infection in China, with other food animals also remaining as threats to public health (Guo et al., 2015; Yang et al., 2017b).

## Sheep

**FIGURE 1:**
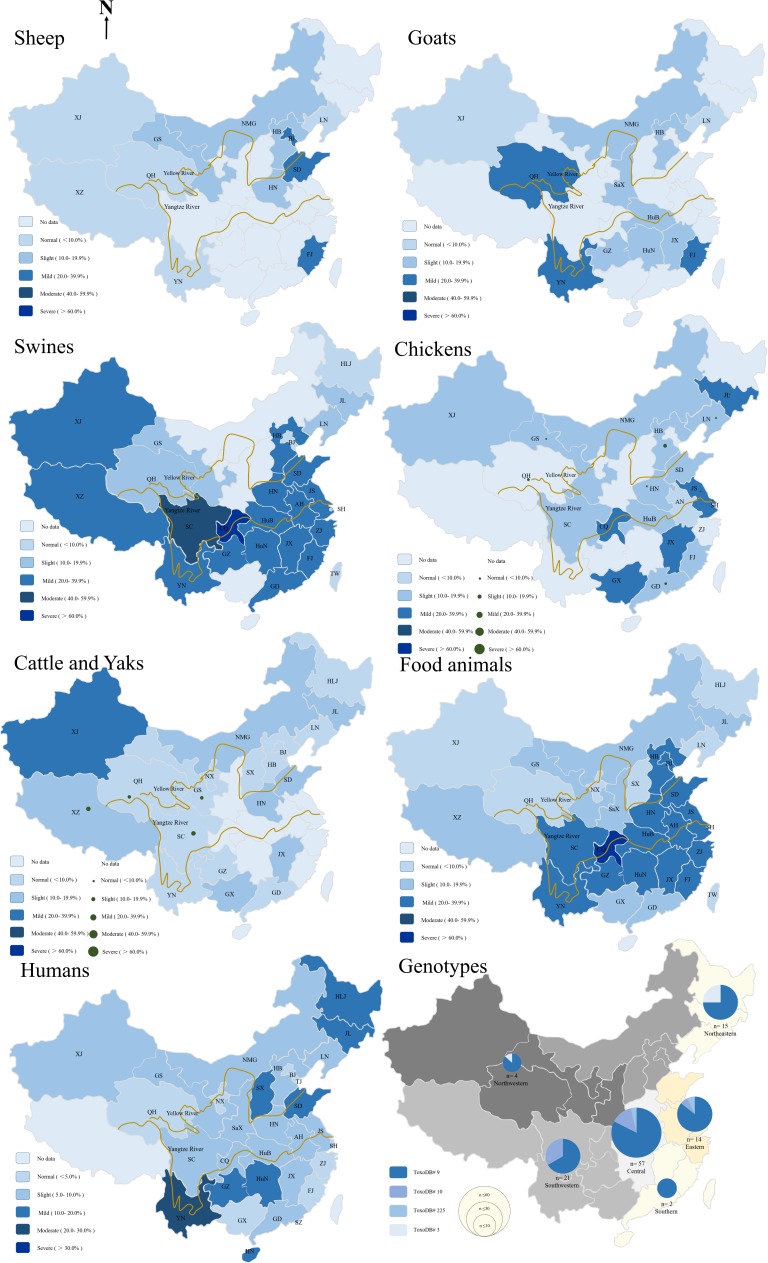
Geographical distribution map of epidemiology of *Toxoplasma gondii* in sheep, goats, swines, chickens, cattle and yaks, food animals summary, humans, and genotypes of *T. gondii* in food animals from 2000–2017 in China. HLJ: HeiLongJiang; JL: JiLin; LN: LiaoNing; HB: HeBei; GS: GanSu; QH: QingHai; XJ: XinJiang; XZ: Tibet; SC: SiChuan; CQ: ChongQing municipality; GZ: GuiZhou; HN: HeNan; HuB: HuBei; HuN: HuNan; SD: ShanDong; JS: JiangShu; ZJ: ZheJiang; AH: AnHui; JX: JiangXi; FJ: FuJian, YN: YunNan; GD: GuangDong; GX: GuangXi; TW: TaiWan; NX: NingXia; NMG: Inner Mongolia; SX: ShanXi; SaX: ShaanXi; SH: ShangHai; BJ: BeiJing.

Sheep and goats are highly susceptible to *T. gondii* infections. Due to the subtropical climate and geographical environment, little sheep were breed in South China, mutton comes mainly from goats. Mutton, viscera, blood, exudates, and milk from sheep and goats that carry *T. gondii* parasites may infect other animals and humans (Dubey et al., 2014; Ding et al., 2017; Tonouhewa et al., 2017). Based on the data and the findings of Yang (Yang et al., 2017b), an epidemiological map of *T. gondii* in China was generated (**Figure 1**). The seroprevalence for *T. gondii* in sheep has been estimated to be 11.8% (95%CI, 11.33–12.23%, 2,305/19,565), which is lower than that in other countries (Dubey, 2010) (**Supplementary Table S1**).

*Toxoplasma gondii* seroprevalence in sheep varies with altitude, year, climate, geographic regions, abortion history, and age. Compared the prevalence of *T. gondii* infections in sheep sampled from 2000 to 2010, it was significantly increased in the period of 2011–2017 (*P* < 0.0001, OR = 1.578). **Figure 1** shows that sheep from eastern coastal locations have the highest prevalence rates, higher than that of sheep from the west part (*P* < 0.0001, OR = 2.509) (**Supplementary Table S2**). The altitude of eastern coastal provinces is lower than western provinces, and most rivers flow from west to east, into the sea. A previous survey suggested that *T. gondii* oocysts could thus be transported via freshwater runoff into the ocean (Dubey, 2010; Cong et al., 2012; Gao et al., 2016). This might explain the high prevalence of *T. gondii* infection in the east compared with that in the west of China. Therefore, the high prevalence rate of *T. gondii* infections in eastern coastal provinces may be related to the accumulation of oocysts in rivers. Furthermore, the other possible reasons may be that residents in eastern part like to keep pet cats because better economic conditions or the *T. gondii* oocysts benefits from the development under warm climate and moist environment in the east. Abortion history, age, and geographical origin were the main risk factors associated with *T. gondii* infections (Yang et al., 2017b).

Hide (2016) reported that high prevalence of *T. gondii* infection in sheep is due to transplacental transmission rather than oocyst original infection. The seroprevalence of *T. gondii* in sheep from different areas in China was within the range of 0.8–39.3%, indicating sheep could be infected with *T. gondii* with oocysts from contaminated environmental or reactivation *T. gondii* cysts in chronic infection sheep during pregnancy. Most cooking methods for mutton meat were boiling for several hours, even overnight boiling for soup, which may effectively inactivate *T. gondii* cysts (Dubey, 2010). Therefore, the seroprevalence of *T. gondii* in sheep is not directly related to the prevalence of viable *T. gondii* in mutton. Lamb and mutton are very popular in certain parts of China (Gansu, Neimenggu, and Xinjiang), where pork may be eschewed for religious or economic reasons. Mutton is the main ingredient of hotpot, wherein the meat is frozen, sliced, and boiled 3 s to 2 min for eating. No reports describing that hotpot could effectively inactivate *T. gondii* were identified.

## Goats

**FIGURE 2:**
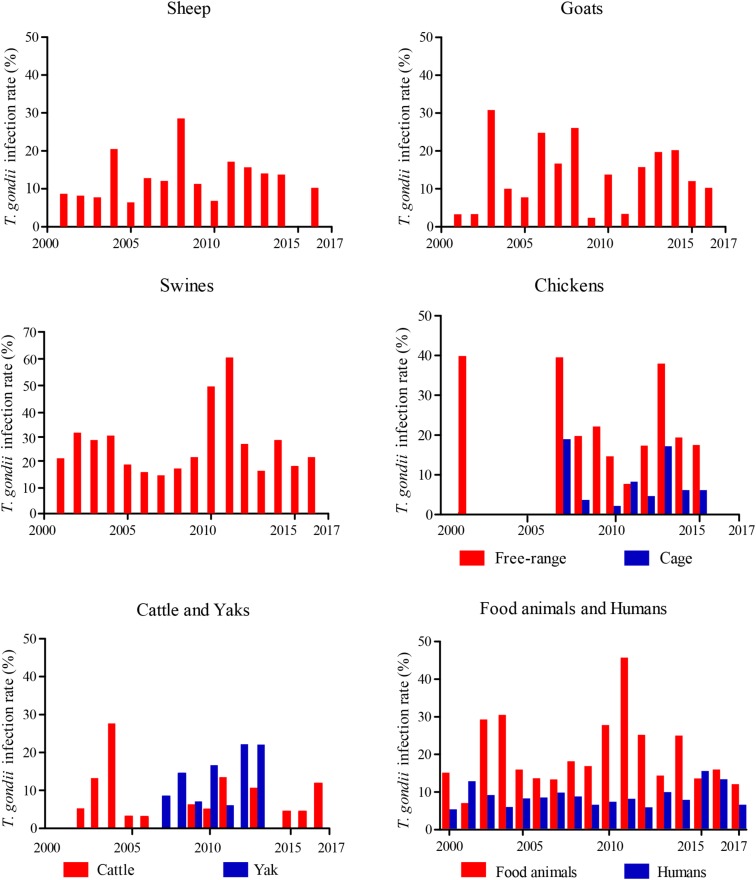
Year distribution map of epidemiology of *Toxoplasma gondii* in food animals and human, from 2000–2017 in China.

*Toxoplasma gondii* could cause abortion and neonatal mortality in goats (Dubey, 2010). Although more information and high prevalence rates of *T. gondii* have been reported in goats from around the world (Dubey, 2010), investigations on cases of toxoplasmosis in goats from China are limited. The overall estimated seroprevalence for *T. gondii* in goats is 17.6% (95%CI, 17.02–18.12%, 3,260/18,556), which is higher than that of sheep (**Figure 1** and **Supplementary Table S3**). In East China, mutton mainly comes from sheep, and very little breeding for goats, so there were few available reports on the prevalence of *T. gondii* in goat from East China. The seroprevalence rates for *T. gondii* infection in goats from south-west of China was higher than other parts (*P* < 0.0001, OR = 1.130). Compared the prevalence of *T. gondii* infection in goats sampled from 2000 to 2010 (19.32%, 95%CI, 18.55–20.12%), the prevalence rate was decreased from 2011 to 2017 (14.09%, 95%CI, 13.38–14.83%) (*P* < 0.0001, OR = 1.460, **Figure 2**). Gender, season, age, geographical origin, the presence of cats, hygiene, and abortion history are risks for *T. gondii* infections in goats. Goat meat and milk contaminated *T. gondii* may have potential threaten for healthy consumers.

## Swines

China is the largest consumer and a global producer of pork. *T. gondii* was isolated from retail pork samples (Dubey et al., 2005; Wang et al., 2012). The overall estimated seroprevalence for *T. gondii* in swine is 32.9% (95%CI, 32.54–33.16%, 29,559/89,978) (**Supplementary Table S4**). The prevalence of *T. gondii* varies widely among sample sources. Overall, the infection of *T. gondii* in swine from free-ranging and animal hospitals is higher than that of farms and slaughterhouses (*P* < 0.0001, OR = 1.201). The pigs from Chongqing were the most severely infected with *T. gondii*, whereas those from Sichuan, Guizhou provinces showed moderate degrees of infection (**Figure 1**). In general, the level of *T. gondii* infection in most areas of China is moderate and may be related to the presence of cats and dogs, the size of farm, age, breeding density, and insects, the frequency of scavenging, and management and transport of pigs (Wang et al., 2012; Liu et al., 2017).

The overall seroprevalence for *T. gondii* in swine is similar to that of American countries (Assadi-Rad et al., 1995; Suarez-Aranda et al., 2000). Further investigation indicated that these geographic surveys for *T. gondii* infections vary in terms of serological testing method used and cutoff values (Dubey, 2010). Nevertheless, a higher prevalence (63.3%) has been reported in Argentina (Omata et al., 1994), and lower seroprevalence rates have been reported in Mexico (8.9%) (Garcia-Vazquez et al., 1993) and Canada (9.4%) (Smith, 1991). In China, compared the prevalence of *T. gondii* infections in swines sampled from 2000 to 2010 (27.58%, 95%CI, 27.13–28.03%), it was significantly increased in the period of 2011–2017 (36.64%, 95%CI, 36.22–37.05%) (*P* < 0.0001, OR = 1.518, **Figure 2**). This may be related to the increased number of cats and the underestimate of *T. gondii* oocysts in the environment.

## Chickens

Viable *T. gondii* strains have been isolated from chickens (Dubey, 2010), and *T. gondii* tachyzoites have been observed in red blood cells of birds (Dubey, 2002). Chickens play a crucial role in the transmission of *T. gondii*, free-ranging chickens are infected with *T. gondii* by ingesting oocysts from soil or food (Feng et al., 2016). Chicken may be utilized as an indicator of environmental and soil contamination with *T. gondii* oocysts. Although chickens are susceptible to *T. gondii* infections, they do not always exhibit clinical symptoms, and only a few reports have described the clinical symptoms of chicken toxoplasmosis. Although the consumption of raw eggs is not significantly associated with *T. gondii* infections (Dubey, 2002), it could not rule out the possibility of infection. However, no direct evidence has been reported.

The estimated seroprevalence for *T. gondii* was 17.9% (95%CI, 16.93–18.83%, 1,116/6,242) in free-ranging chicken, 6.3% (95%CI, 5.63–6.99, 307/4,866) in caged chickens, and overall prevalence in chickens of 19 provinces is 12.8% (95%CI, 12.19–13.43%, 1,423/11,108) (**Supplementary Table S5**). The epidemiological data of *T. gondii* are presented in **Figure 1**. In general, the level of *T. gondii* infection in most areas of China is slight and may be related to the source of chickens; the prevalence of *T. gondii* infections in most free-ranging chickens is higher than that of caged chicken. Compared the prevalence of *T. gondii* infections in chickens sampled from 2000 to 2010 (15.56%, 95%CI, 14.53–16.58%), the prevalence rate was decreased dramatically in the period of 2011–2017 (10.73%, 95%CI, 9.97–11.49%) (*P* < 0.0001, OR = 1.533) (**Figure 2**), which may be related to improved management and intensive farming.

## Cattle and Yaks

Yak (*Bos* gr*unniens*) is a long-haired bovid that is distributed in the Himalayan region of south-central Asia. China has 1.3 million yaks in the Qinghai-Tibetan Plateau, which include wild yak and domestic yak, accounting for 90% of the world’s yak population (Liu et al., 2008; Li et al., 2014). In addition, China is one of the largest countries that engage in cattle farming around the world, raising an estimated 50 million in 2015^1^.

The overall estimated seroprevalence of *T. gondii* in cattle and yak was 9.1% (95%CI, 8.65–9.52%, 1,560/17,168) and 13.5% (95%CI, 12.80–14.21%, 1,221/9,042) respectively from China’s 20 Provinces (**Supplementary Table S6** and **Figure 1**). The overall prevalence of the *T. gondii* was 10.6% (95%CI, 10.24–10.98%, 2,781/26,210) in cattle from China, which is lower than that reported in Estonia 16.8% (743/3991) (*P* < 0.001) (Robert-Gangneux and Darde, 2012), and similar to that reported in Czech Republic 9.7% (53/546) (*P* = 0.5723) (Bartova et al., 2015).

Although cattle are considered as poor hosts for *T. gondii* and good hosts for *N. caninum*, *T. gondii* infections could cause abortion, resulting in substantial economic losses and an increased potential in transmission to other animals and humans (Dubey and Jones, 2008). The observed higher seroprevalence of *T. gondii* in cattle from Xinjiang (Wang et al., 2011) and Qinghai (Liu et al., 2011) may possibly be due to geographical factors such as rivers, rainfall, wild animals, and differences in landscape. Compared to cattle that do not have a history of abortion, *T. gondii* infections are a risk for abortion in cattle from the north, northeast, and central China (Sun et al., 2015).

The cattle and yaks from the pastoral area have higher prevalence rates than those in farms, and yaks had higher prevalence rates than cattle raised in pastures (Dong et al., 2011). Ecological and geographical factors, pregnancies, ages, history of abortion, and breed style (graze, barns) are risks for *T. gondii* infections in cattle. *T. gondii* indirectly infects humans through the consumption of raw or undercooked beef or milk (Zhou et al., 2012; Xu et al., 2015).

## Humans

*T. gondii* widely occurs among the Chinese and its prevalence rate widely varies among provinces (**Supplementary Table S7**). For 2000–2017, the overall seroprevalence of *T. gondii* in Chinese is 8.2% (95%CI, 8.06–8.39%, 8,502/103,383) (**Figure 1**), which is relatively lower than that of France (61.0%), Brazil (84.5%), United States (38.0%), and India (24.0%) (Dubey, 2010). The prevalence rate of *T. gondii* is 8.6% in pregnant women or women diagnosed with gynecological diseases, 16.8% in cancer patients. Furthermore, specific professions (butchers, zookeepers, animal traffickers), low level of education, non-Han people, eating raw and undercooked meat were the most important risk factors. In addition, the prevalence of human *T. gondii* infection increased from West China to East China, which coincides with the incidence of *T. gondii* infection in food animals. Further, consistent with that in food animals, compared the prevalence of *T. gondii* infections in human from 2000 to 2010 (7.49%, 95%CI, 7.29–7.68%), the seroprevalence rate for human *T. gondii* infection significantly increased in the period of 2011–2017 (9.69%, 95%CI, 9.38–10.01%), (*P* < 0.0001, OR = 1.326), which may be related to changes in eating habits (steaks, vegetable salads, and barbecues were more popular), increased proportion of meat in people’s diet, and the increased number of pet cats in the past few decades. A recent review of *T. gondii* infections in China also showed the same trend (Pan et al., 2017).

## Clinical Toxoplasmosis in Naturally Infected Food Animals

Most *T. gondii* infection in food animals from China was subclinical. Cases reports with evidence of toxoplasmosis were only found in swine (He et al., 2001; Liao et al., 2006; Li et al., 2010; Deng et al., 2012), few reports in other food animals. There have been three reports of clinical toxoplasmosis in pregnant sow and in fattening swine from Jiangxi Province (42% infected and 8% died) (He et al., 2001; Deng et al., 2012), Gansu Province (57% infected, 2% died) (Li et al., 2010) and Guangdong Province (33% infected, 2% died) (Liao et al., 2006). The case reports found clinicopathological changes in *T. gondii* infected swines were fever, dyspnea, loss appetite, skin cyanosis, abortions or stillbirths. Pulmonary congestion, lymphadenectasis, liver, heart, and spleen were enlarged and necrosis was observed by postmortem examination and histology inspection. Furthermore, *T. gondii* tachyzoites were found in the smear of tracheal lymph node, lung, liver, and spleen under the light microscope, and *T. gondii* could be recovered from mice inoculated with tissue of died swines or sows (He et al., 2001; Liao et al., 2006; Li et al., 2010; Deng et al., 2012). How most swine acquire infection is unknown, uncooked kitchen garbage, birds, rodent or cats may be the source of infection. It was speculated that the ingestion of *T. gondii* oocysts contaminated feed might contribute to the outbreak of toxoplasmosis (He et al., 2001; Li et al., 2010).

## The Molecular Epidemiology of *T. gondii* in Food Animals and Humans

### Isolation and the Genotypes Distribution of *T. gondii*

*T. gondii* is genetically diverse, exhibiting regional differences around the world (Lehmann et al., 2006; Su et al., 2012). *T. gondii* strains isolated from humans and animals are classified into three clonal lineages, namely, types I, II, and III (Howe and Sibley, 1995). A total of 231 genotypes have been identified around the world, which comprises 1,457 *T. gondii* strains^2^. Among 142 viable *T. gondii* isolates from animals and humans in China, most isolates (85 strains, 69.7%) were derived from the cats (Fu et al., 2015). The prevalence and *T. gondii* isolates from cats in China was previously summarized by Yang et al. (Yang et al., 2015). Three *T. gondii* strains were isolated from sheep and genotyped as ToxoDB#9 (Yang et al., 2017b) and ToxoDB#1 (Chen et al., 1988; Lu et al., 2014). Two *T. gondii* strains were isolated from chickens and were genotyped as ToxoDB#225 and Type I (Zhao et al., 2012; Wang et al., 2015). Forty-five *T. gondii* strains were isolated from swines and genotyped as ToxoDB#9, ToxoDB#3, and ToxoDB#1 (Zhou et al., 2009; Jiang et al., 2013, 2015; Li et al., 2015; Wang et al., 2015, 2016). Seven *T. gondii* strains were isolated from humans and genotyped as ToxoDB#1, ToxoDB#9, ToxoDB#3, ToxoDB#10, ToxoDB#204, and ToxoDB#4 (Fu et al., 2015). The number of *T. gondii* isolates from China is relatively small in terms of the broad land of the country. It may be related to the China predominant genotype ToxoDB#9, the virulence and cyst-forming capability differ in the strains sharing the same genotype (Li et al., 2014; Gao et al., 2017). Furthermore, commercial breeding of experimental animal cats and γ-IFN knockout mice in China is limited. *T. gondii* isolations are thus at a bottleneck, and only virulent strains or those with high rates of cyst formation have been successfully isolated. The genotypes of *T. gondii* from samples of food animals are summarized in **Figure 1**. Currently, a total of 112 samples (31 isolates and 81 DNAs) were characterized (**Supplementary Table S8**). Four genotypes have been identified in China, ToxoDB#9 (*Chinese1*) was the predominant genotype (78.57%), and ToxoDB#9 *T. gondii* also appears in Colombia (Dubey et al., 2007a), Vietnam (Dubey et al., 2007b), and Sri Lanka (Dubey et al., 2007c). ToxoDB#10 (type I) is the second most common genotype (14.29%), whereas ToxoDB#3 (type II variant) (3.58) are rarely detected. These findings indicate that *T. gondii* in food animals from China have limited genetic polymorphisms.

Information on the genotypes of *T. gondii* in food animals from China was summarized up in this article. What factors influence ToxoDB#9 to become the predominant genotype and how did this particular genotype emerge? ToxoDB#3 strains are predominant in Europe yet also occur in Northwest and Northeast China (Chaichan et al., 2017). The atypical genotypes may be evolved from the archetypical lineages of types I, II, and III. Two studies supporting the hypothesis of *Chinese 1* preceded Type II *T. gondii*, both of them sharing a common ancestor (Lorenzi et al., 2016; Bertranpetit et al., 2017). According to the geographical distribution of *T. gondii* strains in China (**Figure 1**), it was showed East–West genotype gradient of *Chinese 1.* The geographical distribution of *T. gondii* genotypes may reflect that the continuum with West China for East China and the circulation of strains though Silk Road or maritime coastal road. More epidemiological studies are required to confirm this hypothesis and to clarify the route of propagation of *T. gondii* genotypes in China.

### The Seroepidemiology of *T. gondii* From China

*Toxoplasma gondii* is responsible for 20.7% of foodborne deaths due to known infectious agents (Ortega, 2006). The distribution and regularity of the epidemiology of *T. gondii* in food animals from China are summarized in **Figure 1**. The overall estimated seroprevalence of *T. gondii* in food animals in China is 23.7% (39,194/165,417, 95%CI, 23.49–23.90%), which include 31 provinces (**Supplementary Table S9**). The Yangtze, Yellow and Pearl River are three of the longest rivers in China and thus are the most important water source for the Chinese. The Yellow River runs from west to east, and the food animals from these regions showed increased seroprevalence for *T. gondii*. Compared the prevalence of *T. gondii* infection in food animals from Yellow River upstream (12.20%, 95%CI, 13.50–14.28%), the seroprevalence rate for *T. gondii* infection were increased in food animals from Yellow River midstream (21.80%, 95%CI, 27.09–28.67%) and downstream (21.40%, 95%CI, 25.37–29.07) (*P* < 0.0001). Meanwhile, during 2000–2010 annual accumulated, Yellow River downstream region maximum, minimum and average precipitation (1090, 456.6, and 693.5 mm) was higher than midstream region (995.3, 349.9, and 621.4 mm) and upstream region (800.4, 348, and 564.4 mm). (^3^National meteorological information center, China).

The Yangtze runs from the west to the east across central China, and food animals from these regions (28.76%, 95%CI, 39.93–40.80%) have higher seroprevalence for *T. gondii* than non-Yangtze River regions (20.37%, 95%CI, 25.27–25.89%), and much prefer Yellow River regions (15.57%, 95%CI, 18.09–18.80%) (*P* < 0.0001, OR = 2.189) and Pearl River regions (25.78%, 95%CI, 34.05–35.43%) (*P* < 0.0001, OR = 1.162). Moreover, food animals from Pearl River regions have higher seroprevalence for *T. gondii* than Yellow River regions (*P* < 0.0001, OR = 1.883). During 2000–2010 annual accumulated, Pearl River region maximum, minimum and average precipitation (2090.9, 1034.4, and 1463.8 mm) was higher than Yangtze River region (1514.7, 713.8, and 1082.7 mm) and Yellow River region (897.5, 364.0, and 599.1 mm). (^3^National meteorological information center, China). Results suggested that *T. gondii* oocysts may be transmitted by water, and annual precipitation possible help the oocyst spread and retain accessible for potential hosts, which in turn has fueled efforts in designing prevention and control strategies, including water filtration or water boiling, inactivating oocysts from felines, and monitoring birds and rodents.

In this paper, an only apparent seroprevalence of *T. gondii* infection from food animals and humans was showed and analyzed, the sensitivity and specificity of serologic assays in different hosts and different serological detection methods were not evaluated. In order to get a clearer picture of the true prevalence of *T. gondii* infection in China, apparent seroprevalence need proceed by Bayesian statistics for all unknown parameters (different samples, different hosts, different serological test methods, and different test kits) in the future (Basanez et al., 2004).

## Conclusion and Perspective

*Toxoplasma gondii* can pose a high risk in immunocompromised individuals for serious health problems and result in destructive consequences. In recently two decades, Chinese scientists have obtained lots of viable *T. gondii* isolates from chickens, swines, sheep, and cats, indicating *T. gondii* is widespread in China. This review collected the data on *T. gondii* infection in food animals (sheep, goats, swine, chickens, cattle) and humans from China, and showing the epidemiological distribution of *T. gondii*. The predominant genotype of *T. gondii* in food animals from China is *Chinese 1* (ToxoDB#9). The overall seroprevalence of *T. gondii* infections in food animals is 23.7%, which is three times of that in humans. The seroprevalence of *T. gondii* infections in animals and humans was significantly increased in the period of 2011–2017 when compared with data from 2000 to 2010 (*P* < 0.0001). Further, food animals from regions of the Yangtze River have higher seroprevalence rates for *T. gondii* than ones from non-Yangtze River (*P* < 0.0001), suggesting *T. gondii* oocysts may be transmitted by water and annual precipitation possible help the oocyst spread and retain accessible for potential hosts. This suggestion was confirmed by data within the Yellow River regions, where an increasing trend in seroprevalence for *T. gondii* (*P* < 0.0001) was found from upstream western to midstream and downstream eastern regions. Therefore, effective prevention and control strategies are proposed to include water filtration or water boiling, inactivating oocysts from feline’s feces, monitoring birds and rodents.

## Author Contributions

YY conceived and designed the review. HD and YY drafted the manuscript. All authors contributed to the writing of the manuscript, critically reviewed the draft, and approved the final version of the manuscript.

## Conflict of Interest Statement

The authors declare that the research was conducted in the absence of any commercial or financial relationships that could be construed as a potential conflict of interest.

## Footnotes

1 http://data.stats.gov.cn/

2 http://ToxoDB.org

3 http://data.cma.cn/
