# Supplementary material for: Prevalence, Risk Factors, and Genotypes of Toxoplasma gondii in Food Animals and Humans (2000–2017) From China
Source: Front Microbiol. 2018 Sep 11;9:2108. doi: 10.3389/fmicb.2018.02108 (PMC6141634; doi:10.3389/fmicb.2018.02108)
Supplement: Supplementary file 1 [file Table_1.docx]

**Web extra material**

**Table S1 The seroprevalence of *T. gondii* in food animals from the world^1^**

| **Animal** | **North America** | **Africa** | **South America** | **Europe** | **Oceania** | **Asia(excluding China)** | **China** |
| --- | --- | --- | --- | --- | --- | --- | --- |
| **Sheep** | 54.6% (4733/8676) | 26.6% (1243/4674) | 30.0% (2590/8647) | 38.3% (12373/32344) | — | 33.9% (3877/11436) | 11.8% (2305/19565) |
| **Goat** | 19.4% (393/2026) | 35.9% (2878/8027) | — | 30.1% (2107/6988) | 25.2%(75/298) | 21.3% (1346/6311) | 17.6% (3260/18556) |
| **Swine** | 12.9% (11061/86041) | 32.6% (341/1046) | 29.1% (1537/5288) | 6.3% (3698/58964) | — | 21.4% (1920/8985) | 32.9% (29559/89978) |
| **Chicken** | 38.4% (310/808) | 39.1% (210/537) | 52.6% (501/953) | 33.0% (380/1150) | — | 25.6% (394/1542) | 12.8% (1423/11108) |
| **Cattle** | 11.0% (50/456) | 6.6% (52/785) | 41.6% (2107/5067) | 13.4% (2679/19948) | — | 13.7% (1146/8361) | 10.6% (2781/26210) |
| **Human** | 46.0% (15999/34808) | 58.0% (9619/16595) | 54.1% (66866/123555) | 44.5% (219346/493055) | — | 28.0% (11952/42655) | 8.2% (8502/103383) |

**Table S2 Supplement data* on the seroprevalence of *T. gondii* infection in sheep (2000–2017)**

| **Area** | **Province** | **Year** | **Source** | **Method, Titer** | **No. tested** | **No. positive** | **Positive (%)** | **Risk** | **References** |
| --- | --- | --- | --- | --- | --- | --- | --- | --- | --- |
| **North China** | **HeBei** | <2006 | Slaughter house | — | 442 | 17 | 3.85% | — | ^2^ |
|  | **Inner Mongolia** | 2010 | Grazing | MAT, 1:25 | 263 | 28 | 10.65% | — | ^3^ |
| **Southwest China** | **YunNan** | 2012-2013 | — | IHA^c^, 1:16 | 154 | 15 | 9.74% | Geographical origin | ^4^ |
| **Northwest China** | **GanSu** | <2009 | Domestic | IHA^c^, 1: 16 | 885 | 161 | 18.19% | — | ^68^ |
|  |  | 2011 | Veterinary Station | IHA^c^, 1: 64 | 130 | 8 | 6.15% | Breeding system, management, animal welfare | ^6^ |
|  | **QingHai** | <2001 | Slaughter house | IHA^c^, — | 518 | 4 | 0.77% | — | ^7^ |
|  |  | 2007 | — | IHA^c^, 1: 64 | 692 | 54 | 7.80% | — | ^8^ |
|  |  | 2009 | Countryside | ELISA^c^ | 110 | 14 | 12.73% | — | ^9^ |
|  |  | <2010 | — | IHA^c^, 1: 64 | 100 | 4 | 4.00% | — | ^10^ |
|  |  | <2010 | — | ELISA^c^ | 2402 | 56 | 2.33% | — | ^11^ |
|  |  | 2010 | — | IHA^—^, 1:64 | 32 | 10 | 31.25% | — | ^12^ |
|  |  | <2011 | Countryside | IHA^c^, 1: 64 | 670 | 182 | 27.16% | — | ^13^ |
|  |  | 2010 | Domestic | ELISA^o^ | 220 | 11 | 5.00% | — | ^14^ |
|  |  | 2013 | — | IHA^c^, 1: 64 | 282 | 60 | 21.28% | — | ^15^ |
|  |  | 2013 | Countryside | IHA^c^, 1: 64 | 199 | 9 | 4.52% | — | ^16^ |
|  |  | 2013 | — | IHA^c^, 1: 64 | 204 | 5 | 2.45% | — | ^17^ |
|  |  | 2013 | — | IHA^c^, 1: 64 | 201 | 4 | 1.99% | — | ^18^ |
|  |  | 2014 | Grazing | IHA^c^, 1: 64 | 200 | 12 | 6.00% | — | ^19^ |
|  |  | 2016 | Grazing | IHA^c^, 1: 16 | 350 | 17 | 4.86% | — | ^20^ |
|  | **XinJiang** | 2005 | — | IHA^c^, 1: 64 | 140 | 9 | 6.43% | — | ^21^ |
|  |  | <2012 | Domestic | IHA^c^, — | 50 | 8 | 16.00% | — | ^22^ |
|  |  | <2014 | Grazing | IHA^c^, — | 565 | 37 | 6.55% | — | ^5^ |
| **Northeast China** | **LiaoNing** | 2012 | — | MAT, 1:25 | 402 | 72 | 17.91% | Age, Gender, Location, Size, Rearing system | ^44^ |

***:** Based on the data from Yang ^229^.

Note: There was no data from South China.

**Table S3 Seroprevalence of *T. gondii* infection in goats (2000–2017)**

| **Area** | **Province** | **Year** | **Source** | **Method, Titer** | **No. tested** | **No. positive** | **Positive (%)** | **Risk studies** | **References** |
| --- | --- | --- | --- | --- | --- | --- | --- | --- | --- |
| **East China** | **FuJian** | 2013 | Grazing | IHA^c^, 1:64 | 210 | 43 | 20.48% | — | ^23^ |
|  | **JiangXi** | 2016 | Farms | IHA^c^, 1:64 | 340 | 35 | 10.29% | — | ^24^ |
| **North China** | **HeBei** | <2004 | Slaughter house  Free-ranging, Farm | IHA^c^, 1:64 | 100 | 10 | 10.00% | — | ^25^ |
|  | **Inner Mongolia** | <2006 | Domestic | IHA^c^, 1:64 | 149 | 37 | 24.83% | — | ^26^ |
|  |  | 2010 | Grazing | MAT, 1:25 | 242 | 19 | 7.85% | — | ^3^ |
| **Central China** | **HuBei** | 2006-2008 | Animal hospital | MAT, — | 60 | 16 | 26.67% | — | ^27^ |
|  | **HuNan** | 2014-2015 | Domestic | IHA^c^, 1:64 | 1028 | 124 | 12.06% | Female, Autumn, Age | ^28^ |
| **Southwest China** | **GuiZhou** | 2011-2012 | Domestic | IHA^c^, — | 475 | 6 | 1.26% | — | ^29^ |
|  |  | <2012 | Domestic | IHA^c^, 1:64 | 195 | 2 | 1.03% | — | ^30^ |
|  |  | 2012 | Domestic | IHA^c^, 1:64 | 2840 | 593 | 20.88% | — | ^31^ |
|  | **YunNan** | <2003 | Domestic | IHA^c^, 1: 64 | 3925 | 1210 | 30.83% | Abortion | ^32^ |
|  |  | 2012-2013 | — | IHA^c^, 1:64 | 392 | 69 | 17.60% | Geographical origin | ^4^ |
|  |  | 2013 | Countryside | IHA^c^, 1:64 | 427 | 27 | 6.32% | — | ^33^ |
| **Northwest China** | **QingHai** | 2001 | Domestic | IHA^c^, 1:64 | 602 | 20 | 3.32% | — | ^34^ |
|  |  | 2005-2009 | Domestic | IHA^c^, 1:64 | 211 | 5 | 2.37% | — | ^35^ |
|  |  | <2007 | Domestic | IHA^c^, 1:64 | 527 | 88 | 16.70% | — | ^36^ |
|  |  | <2008 | Veterinary station | IHA^m^, 1:64 | 1028 | 268 | 26.07% | — | ^37^ |
|  |  | 2010 | — | IHA^c^, 1:64 | 178 | 36 | 20.22% | — | ^12^ |
|  |  | <2011 | Countryside | IHA^a^, 1: 64 | 140 | 2 | 1.43% | — | ^13^ |
|  |  | 2012-2013 | Domestic | ELISA^p^ | 650 | 192 | 29.54% | Cats, Hygiene | ^142^ |
|  | **ShaanXi** | 2010 | Domestic | IHA^c^, 1:64 | 751 | 106 | 14.11% | Male, Age | ^39^ |
|  |  | 2013-2014 | Domestic | IHA^c^, 1:64 | 332 | 99 | 29.82% | Male, Age, Location | ^40^ |
|  | **XinJiang** | <2002 | Veterinary station | IHA^c^, 1:64 | 1850 | 62 | 3.35% | — | ^41^ |
|  |  | 2005 | — | IHA^c^, 1:64 | 154 | 12 | 7.79% | — | ^21^ |
|  |  | <2014 | Grazing | ELISA^m^ | 524 | 74 | 14.12% | Abortion | ^42^ |
| **Northeast China** | **LiaoNing** | 2011 | Countryside | IHA^c^, 1:64 | 360 | 15 | 4.17% | — | ^43^ |
|  |  | 2012 | — | IHA^c^, 1:64 | 650 | 58 | 8.92% | Age, Female,  Geographical origin | ^38^ |
|  |  | 2012 | — | MAT, 1:25 | 216 | 32 | 14.81% | Gender, Location, Size, Rearing system | ^44^ |

**Table S4 Seroprevalence of *T. gondii* infection in swine (2000–2017)**

| **Area** | **Province** | **Year** | **Source** | **Method, Titer** | **No. tested** | **No. positive** | **Positive (%)** | **Risk analysis** | **References** |
| --- | --- | --- | --- | --- | --- | --- | --- | --- | --- |
| **East China** | **AnHui** | 2003 | Slaughter house | IHA^c^, 1: 64 | 1050 | 286 | 27.24% | — | ^45^ |
|  |  | <2011 | Farm | IHA^b^, 1: 64 | 1227 | 298 | 24.29% | — | ^46^ |
|  |  | 2011 | Rtail meat stores | ELISA^a^ | 416 | 42 | 10.10% | — | ^47^ |
|  | **FuJian** | <2005 | Free-ranging, farm | IHA^c^, 1: 64 | 1169 | 280 | 23.95% | Age, Seasons | ^48^ |
|  |  | <2006 | Free-ranging, farm | IHA^c^, 1: 64 | 180 | 47 | 26.11% | — | ^49^ |
|  |  | 2006-2007 | Farm, Slaughter house | IHA^c^, 1: 64 | 605 | 87 | 14.38% | Seasons, Geographical origin | ^50^ |
|  | **JiangSu** | 2003 | Slaughter house | IHA^c^,1:64 | 1710 | 414 | 24.21% | — | ^45^ |
|  |  | <2004 | Farm | IHA^c^, 1: 64 | 2408 | 792 | 32.89% | — | ^51^ |
|  | **JiangXi** | <2014 | Domestic | IHA^a^, 1: 64 | 1232 | 282 | 22.89% | Geographical location, Age, Sampling season | ^52^ |
|  | **ShanDong** | 2003 | Slaughter house | IHA^c^, 1: 64 | 1100 | 344 | 31.27% | — | ^45^ |
|  | **ShangHai** | 2008-2009 | Farm, Slaughter house | IHA ^b^, 1: 64 | 882 | 67 | 7.60% | — | ^53^ |
|  | **TaiWan** | 2003-2004 | Slaughter house | LAT^l^, 1:32 | 395 | 36 | 9.11% | — | ^54^ |
|  | **ZheJiang** | 2003 | Slaughter house | IHA^c^, 1: 64 | 780 | 236 | 30.26% | — | ^45^ |
|  |  | 2008-2012 | Free-ranging, Farm | ELISA^-^ | 15564 | 4420 | 28.40% | Seasons | ^55^ |
|  |  | 2009-2010 | Farm | ELISA^i^ | 813 | 434 | 53.38% | Size, Age, Geographical origin | ^56^ |
|  |  | <2011 | Veterinary hospital | Test paper ^h^ | 147 | 38 | 25.85% | — | ^57^ |
| **North China** | **BeiJing** | <2006 | Farm | — | 326 | 0 | 0 | — | ^2^ |
|  |  | <2009 | Farm | IHA^c^, 1: 64 | 523 | 49 | 9.37% | Age, Sow, Cats | ^58^ |
|  | **HeBei** | 2000-2001 | Farm, Free-ranging | ELISA, — | 327 | 108 | 33.03% | — | ^59^ |
|  |  | <2004 | Slaughter house, Free-ranging, Farm | IHA^c^, 1: 64 | 1300 | 464 | 35.69% | Age | ^60^ |
|  |  | <2005 | Slaughter house | IHA^c^, 1: 64 | 320 | 26 | 8.13% | — | ^61^ |
|  |  | 2008-2009 | Farm | ELISA | 3558 | 873 | 24.54% | Cats, Breeding Density, Insects, Frequency of scavenge, Age | ^62^ |
| **Central China** | **HeNan** | 2003 | Slaughter house | IHA^c^, 1: 64 | 900 | 316 | 35.11% | — | ^45^ |
|  |  | 2004 | Free-ranging, Farm | IHA^c^, 1: 64 | 65 | 7 | 10.77% | — | ^63^ |
|  |  | 2005 | Hospital | IHA^c^, 1: 64 | 492 | 65 | 13.21% | Geographical origin, Seasons | ^64^ |
|  |  | <2006 | Farm | IHA^c^ 1: 64 | 889 | 147 | 16.54% | — | ^65^ |
|  |  | <2008 | Farm, Veterinary station | IHA^c^, 1: 64 | 2325 | 304 | 13.08% | — | ^66^ |
|  |  | <2009 | Farm | IHA^c^, 1: 64 | 897 | 159 | 17.73% | Age | ^67^ |
|  |  | 2011-2013 | Farm | IHA^c^, 1: 64 | 428 | 109 | 25.47% | Cats, Mice, Dogs, History of abortion | ^112^ |
|  |  | 2014 | Free-ranging, Farm | ELISA^f^ | 4680 | 1635 | 34.94% | Geographical origin, Age, | ^69^ |
|  | **HuBei** | 2006-2008 | Animals hospital | MAT, — | 197 | 72 | 36.55% | — | ^27^ |
|  |  | 2009-2010 | Farm | ELISA | 2277 | 673 | 29.56% | Cat density | ^70^ |
|  |  | 2008-2009 | Farm | ELISA | 3558 | 873 | 24.54% | Cats, Breeding Density, Insects, frequency of Scavenge, Age | ^62^ |
|  | **HuNan** | 2010-2012 | Farm | IHA^c^,1:64 | 1191 | 373 | 31.32% | Seasons | ^71^ |
|  |  | 2015-2016 | Free-ranging, farm | ELISA^g^ | 1302 | 351 | 26.96% | Source | ^72^ |
| **South China** | **GuangDong** | <2006 | Farm | IHA^c^, 1: 64 | 269 | 61 | 22.68% | — | ^73^ |
|  |  | 2008-2009 | — | ELISA^j^ | 1022 | 276 | 27.01% | Types of pig, Geographical origin | ^74^ |
| **Southwest China** | **ChongQing** | 2004-2011 | Slaughterhouse, Free-ranging, Farm | ELISA^d^ | 11700 | 8886 | 75.95% | Years, Source | ^75^ |
|  |  | <2008 | Free-ranging, Farm | ELISA^e^ | 283 | 171 | 60.42% | — | ^76^ |
|  |  | <2012 | Slaughterhouse | IHA^b^, 1:64 | 908 | 278 | 30.62% | — | ^77^ |
|  |  | 2013 | Farm | IHA^c^, 1: 64 | 1109 | 100 | 9.02% | Age, [Management](javascript:void(0);) | ^78^ |
|  | **GuiZhou** | <2010 | Free-ranging, farm | ELISA^e^ | 2906 | 1913 | 65.83% | — | ^79^ |
|  |  | 2011-2012 | Slaughterhouse | ELISA^e^ | 70 | 49 | 70.00% | — | ^80^ |
|  |  | 2012-2013 | Free-ranging, Farm, unhealthy | IHA^c^, 1: 64 | 1494 | 240 | 16.06% | Source | ^81^ |
|  |  | 2014-2015 | Free-ranging, Farm | IHA^c^, 1: 64 | 984 | 171 | 17.38% | Source | ^82^ |
|  |  | <2016 | Free-ranging, Farm | IHA^c^, — | 925 | 156 | 16.86% | — | ^83^ |
|  | **SiChuan** | 2010 | Farm | ELISA^k^ | 365 | 165 | 45.21% | Age | ^84^ |
|  | **Tibet** | 2010 | Free-ranging | MAT,1:25 | 427 | 97 | 22.72% | Types of pig | ^85^ |
|  | **YunNan** | <2002 | — | IHA^b^, 1: 64 | 235 | 77 | 32.77% | — | ^86^ |
|  |  | 2008-2009 | Slaughter house, Farm | IHA^b^, 1: 64 | 831 | 141 | 16.97% | — | ^87^ |
|  |  | 2009 | Free-ranging, Farm | IHA^b^, 1: 64 | 711 | 174 | 24.47% | Source | ^88^ |
|  |  | 2011 | Free-ranging | IHA^c^, 1: 64 | 42 | 9 | 21.43% | — | ^89^ |
| **Northwest China** | **GanSu** | <2001 | Slaughter house, Farm | IHA^c^, — | 214 | 7 | 3.27% | — | ^7^ |
|  |  | 2015 | Free-ranging, farm | IHA^c^, 1: 64 | 550 | 91 | 16.55% | — | ^90^ |
|  | **QingHai** | <2003 | Free-ranging, Farm | IHA^c^, 1: 64 | 251 | 4 | 1.59% | — | ^91^ |
|  |  | <2003 | — | IHA^c^, 1: 64 | 200 | 58 | 29.00% | — | ^92^ |
|  |  | 2003-2004 | Farm | IHA^c^, 1: 64 | 139 | 10 | 7.19% | — | ^93^ |
|  |  | 2007 | Free-ranging, Farm | IHA^c^, 1: 64 | 348 | 41 | 11.78% | — | ^8^ |
|  |  | <2008 | Slaughter house | IHA^c^, — | 288 | 10 | 3.47% | — | ^7^ |
|  |  | <2008 | Veterinary station | IHA^m^, 1: 64 | 80 | 11 | 13.75% | — | ^37^ |
|  |  | 2013-2014 | Farm | ELISA^j^ | 462 | 164 | 35.50% | Age, Seasons | ^94^ |
|  | **XinJiang** | 2010 | Farm | ELISA^a^ | 900 | 327 | 36.33% | — | ^95^ |
|  |  | <2011 | Veterinary station | IHA^c^, 1: 64 | 456 | 139 | 30.48% | — | ^96^ |
|  |  | 2011 | Farm or Free-ranging | ELISA^a^ | 667 | 315 | 47.23% | Geographical origin | ^97^ |
|  |  | <2016 | Farm, Free-ranging | ELISA^g^ | 433 | 55 | 12.70% | Source | ^98^ |
| **Northeast China** | **HeiLongJiang** | 2011-2012 | Farm | IHA^c^, 1: 64 | 1014 | 47 | 4.64% | Source, Geographical origin | ^99^ |
|  | **JiLin** | 2013 | Farm, Slaughter house | IHA ^b^, 1: 64 | 1235 | 236 | 19.11% | Geographical origin, Types of pig | ^100^ |
|  | **LiaoNing** | 2011 | Slaughter house | IHA ^c^,1:64 | 1164 | 140 | 12.03% | Geographical origin | ^101^ |
|  |  | 2013-2014 | Slaughter house | MAT, 1: 25 | 2063 | 233 | 11.29% | — | ^102^ |

**Table S5** **Seroprevalence of *T. gondii* infection in chicken (2000–2017)**

| **District** | **Area** | **Year** | **Source** | **Method, Titer** | **No. tested** | **No. positive** | **Positive (%)** | **Risk analysis** | **References** |
| --- | --- | --- | --- | --- | --- | --- | --- | --- | --- |
| **East China** | **AnHui** | 2010 | Free-range | ELISA^r^ | 60 | 0 | 0 | — | ^103^ |
|  | **FuJian** | 2010 | Free-range | ELISA^r^ | 64 | 10 | 15.63% | — | ^103^ |
|  | **JiangSu** | 2009-2010 | Free-range | ELISA^r^ | 165 | 58 | 35.15% | Season | ^103^ |
|  |  | <2012 | Free-range | ELISA^r^ | 309 | 53 | 17.15% | Source | ^104^ |
|  |  |  | Caged |  | 150 | 4 | 2.67% |  |  |
|  | **JiangXi** | 2010 | Free-range | ELISA^r^ | 111 | 25 | 22.52% | — | ^103^ |
|  | **ShanDong** | 2009 | Free-range | ELISA^r^ | 71 | 9 | 12.68% | — | ^103^ |
|  | **ShangHai** | <2015 | Caged | IHA^c^, 1: 64 | 95 | 1 | 1.05% | Source | ^105^ |
|  |  |  | Free-range |  | 234 | 32 | 13.68% |  |  |
| **North China** | **HeBei** | 2000-2001 | Free-range | ELISA^s^ | 413 | 162 | 39.23% | — | ^59^ |
|  |  | <2010 | Free-range | IHA^c^, 1:64 | 345 | 38 | 11.01% | — | ^106^ |
|  |  |  | Caged |  | 235 | 5 | 2.13% |  |  |
|  |  | <2010 | Free-range | IHA^c^, 1: 64 | 364 | 24 | 6.59% | — | ^107^ |
|  |  |  | Caged |  | 120 | 0 | 0 |  |  |
|  | **Inner Mongolia** | 2010 | Free-range | ELISA^r^ | 61 | 7 | 11.48% | — | ^108^ |
| **Central China** | **HuBei** | 2007-2008 | Free-range | LAT^t^ | 95 | 37 | 38.95% | Source | ^109^ |
|  |  |  | Caged |  | 191 | 35 | 18.32% |  |  |
|  |  | 2010-2016 | Wilds chickens | IHA^c^, 1:64 | 571 | 72 | 12.61% | Gender | ^110^ |
|  |  | 2011-2012 | Caged | IHA^c^, 1:64 | 400 | 77 | 19.25% | Geographical origin, Feeding style, Source | ^111^ |
|  |  |  | Free-range |  | 296 | 81 | 27.36% |  |  |
|  | **HeNan** | 2010 | Free-range | ELISA^r^ | 135 | 17 | 12.59% | Source | ^103^ |
|  |  |  | caged |  | 93 | 2 | 2.15% |  |  |
|  |  | 2011-2013 | caged | IHA^c^, 1: 64 | 551 | 31 | 5.63% | Year | ^112^  ^232^ |
|  |  | 2015 | Free-range | MAT, 1:25 | 700 | 132 | 18.86% | Gender |  |
| **South China** | **GuangDong** | 2008 | Free-range | MAT, 1: 20 | 361 | 21 | 5.82% | — | ^113^ |
|  |  |  | Caged |  | 244 | 3 | 1.23% |  |  |
|  |  | 2010 | Free-range | ELISA^r^ | 72 | 14 | 19.44% | — | ^103^ |
|  |  | <2013 | Free-range | IHA^c^, 1: 64 | 83 | 31 | 37.35% | Source | ^114^ |
|  |  |  | Caged |  | 380 | 63 | 16.58% |  |  |
|  | **GuangXi** | 2009 | Free-range | ELISA^r^ | 140 | 39 | 27.86% | — | ^103^ |
| **Southwest China** | **ChongQing** | 2010 | Free-range | ELISA^r^ | 84 | 25 | 29.76% | — | ^108^ |
|  | **SiChuan** | 2009 | Free-range | ELISA^r^ | 93 | 11 | 11.83% | — | ^103^ |
| **Northwest China** | **Gansu** | 2011 | Free-range | IHA^c^, 1:64 | 92 | 9 | 9.78% | Source | ^6^ |
|  |  |  | Caged |  | 187 | 6 | 3.21% |  |  |
|  |  | 2011 | Free-range | MAT, 1:20 | 108 | 1 | 0.93% | — | ^115^ |
|  |  |  | Caged |  | 305 | 2 | 0.66% |  |  |
|  |  | 2015 | Caged | IHA^c^, 1: 64 | 605 | 10 | 1.65% | — | ^90^ |
|  | **QingHai** | <2008 | Veterinary station | IHA^m^, 1: 64 | 66 | 7 | 10.61% | — | ^37^ |
|  | **XinJiang** | 2010  <2015 | Free-range | ELISA^r^ | 67 | 7 | 10.45% | — | ^103^ |
|  |  |  | Free-range | IHA^c^, 1: 64 | 100 | 12 | 12.00% | — | ^116^ |
| **Northeast China** | **LiaoNing** | 2010 | Free-range | ELISA^r^ | 50 | 4 | 8.00% | — |  |
|  |  | 2011-2012 | Free-range | MAT,— | 110 | 11 | 10.00% | Source | ^117^ |
|  |  |  | Caged |  | 392 | 13 | 3.32% |  |  |
|  |  | 2014 | Free-range | MAT, 1:25 | 206 | 23 | 11.17% | Source | ^118^ |
|  |  |  | Caged |  | 296 | 14 | 4.73% |  |  |
|  |  | <2014 | Free-range | MAT, 1:20 | 160 | 30 | 18.75% | Age, Source, Species | ^119^ |
|  |  |  | Caged |  | 450 | 25 | 5.56% |  |  |
|  | **JiLin** | <2008 | Free-range | ELISA^m^ | 308 | 107 | 34.74% | — | ^120^ |
|  |  |  | Caged |  | 210 | 6 | 2.86% |  |  |
|  |  | <2015 | Free-range | ELISA^m^ | 110 | 17 | 15.45% | — | ^121^ |

**Table S6 Seroprevalence of *T. gondii* infection in cattle and yaks (2000–2017)**

| **District** | **Area** | **Year** | **Source** | **Method, Titer** | **No. tested/ No. positive**  **(Positive %)** | | | **Risk studies** | **References** |
| --- | --- | --- | --- | --- | --- | --- | --- | --- | --- |
| **Cattle** | | | | | | | | | |
| **East China** | **JiangXi** | 2017 | Farms | IHA^c^, 1: 64 | 35 | 4 | 11.43% | — | ^24^ |
|  | **ShanDong** | 2005 | Veterinary hospital | IAT^c^, — | 20 | 0 | — | — | ^122^ |
|  |  | ＜2013 | Farm | MAT | 557 | 100 | 17.95% | — | ^123^ |
|  |  | 2013-2014 | Farm | IHA^c^, 1: 64 | 813 | 80 | 9.84% | Age, Gender, Geographical origin, species, season, abortion history, Source of water, Presence of felids | ^124^ |
| **North China** | **BeiJing** | 2005 | Veterinary hospital | IAT^c^, — | 32 | 0 | — | — | ^122^ |
|  | **TianJin** | 2005 | Veterinary hospital | IAT^c^, — | 29 | 0 | — | — | ^122^ |
|  | **HeBei** | ＜2004 | Slaughter house, Free-ranging, Farm | IHA^c^, 1: 64 | 30 | 8 | 26.67% | — | ^25^ |
|  |  | ＜2005 | Farm | IHA^c^, 1: 64 | 68 | 3 | 4.41% | — | ^61^ |
|  |  | 2013-2014 | Farm | IHA^c^, 1: 64 | 712 | 60 | 8.43% | Age, Gender, Geographical origin, species, season, abortion history, Source of water, Presence of felids | ^124^ |
|  |  | ＜2015 | Farm | IHA, — | 200 | 8 | 4.00% | — | ^125^ |
|  | **Inner Mongolia** | 2013-2014 | Farm | IHA^c^, 1: 64 | 957 | 117 | 12.23% | Age, Gender, Geographical origin, species, season, abortion history, Source of water, Presence of felids | ^124^ |
|  |  | 2013-2014 | — | IHA^c^, 1: 64 | 49 | 0 | — | — | ^126^ |
|  | **ShanXi** | 2005 | Veterinary hospital | IAT^c^, — | 26 | 1 | 3.85% | — | ^122^ |
| **Central China** | **HeNan** | 2011-2012 | Farm | Test paper^e^ | 800 | 43 | 5.38% | — | ^127^ |
|  |  | 2011-2013 | Farm | IHA^b^, 1: 64 | 393 | 79 | 20.10% | Year | ^112^ |
|  |  | 2013-2014 | — | IHA^c^, 1: 64 | 102 | 0 | — | — | ^126^ |
| **South China** | **GuangXi** | 2005 | Veterinary hospital | IAT^c^, — | 28 | 0 | — | — | ^122^ |
|  |  | 2009-2010 |  | IHA^c^, 1: 64 | 875 | 120 | 13.71% | Age, Geographical origin, No. pregnancies | ^128^ |
|  | **GuangDong** | 2005 | Veterinary hospital | IAT^c^, - | 18 | 0 | — | — | ^122^ |
|  |  | 2009-2010 | Farms | IHA kit^b^,  1: 64 | 350 | 20 | 5.71% | Age, No. pregnancies | ^129^ |
| **Southwest China** | **SiChuan** | 2005 | Veterinary hospital | IAT^c^, - | 42 | 2 | 4.76% | — | ^122^ |
|  | **Tibet** | 2013 | — | IHA^c^, 1: 64 | 116 | 14 | 12.07% |  | ^130^ |
|  | **YunNan** | ＜2002 | - | IHA^c^, 1: 64 | 95 | 8 | 8.42% | — | ^86^ |
| **Northwest China** | **GanSu** | 2013 | Farms | MAT, 1: 100 | 751 | 13 | 1.73% | Age, Geographical origin, No. pregnancies | ^131^ |
|  |  | 2016 | — | IHA^c^, 1: 64 | 275 | 14 | 5.09% | — | ^6^ |
|  | **NingXia** | 2013 | Farms | MAT,1: 100 | 906 | 67 | 7.40% | Age, Geographical origin, No. pregnancies | ^131^ |
|  | **QingHai** | <2003 | — | IHA^c^, 1: 64 | 150 | 19 | 12.67% | — | ^92^ |
|  |  | ＜2009 | — | ELISA^o^ | 563 | 17 | 3.02% | — | ^132^ |
|  |  | 2010 | Countryside | IHA^c^, 1: 64 | 106 | 4 | 3.77% | — | ^133^ |
|  |  | 2010 | — | IHA^c^, 1: 64 | 47 | 3 | 6.38% | Age, breed | ^134^ |
|  |  | ＜2011 | Grazing and farms | IHA^c^, 1: 64 | 501 | 11 | 2.20% | — | ^13^ |
|  |  | 2016 | Farm | IHA^c^, 1: 64 | 400 | 9 | 2.25% | — | ^135^ |
|  | **XinJiang** | ＜2002 | Veterinary station | IHA^c^, 1: 64 | 160 | 4 | 2.50% | — | ^41^ |
|  |  | 2005 | Veterinary hospital | IAT^c^, — | 37 | 2 | 5.41% | — | ^122^ |
|  |  | ＜2006 | — | IHA^c^, 1: 64 | 150 | 4 | 2.67% | — | ^136^ |
|  |  | ＜2011 | Free range | IHA^c^, 1: 64 | 390 | 181 | 46.41% | — | ^96^ |
|  |  | 2013 | Farm | IHA^c^, — | 660 | 101 | 15.30% | Age, Geographical origin | ^137^ |
| **Northeast China** | **HeiLongJiang** | 2005 | Veterinary hospital | IAT^c^, — | 30 | 1 | 3.3% | — | ^122^ |
|  |  | 2009-2011 | Farm | IHA^b^ 1: 64 | 1803 | 46 | 2.55% | Age, Gender, Rearing system | ^138^ |
|  |  | 2013-2014 | Farm | IHA^c^, 1: 64 | 694 | 80 | 11.53% | Age, Gender, Geographical origin, species, season, abortion history, Source of water, Presence of felids | ^124^ |
|  | **JiLin** | 2011 | Slaughter house | ELISA^d^ | 1040 | 133 | 12.79% | — | ^139^ |
|  |  | 2013-2014 | Farm | IHA^c^, 1: 64 | 638 | 72 | 11.29% | Age, Gender, Geographical origin, species, season, abortion history, Source of water, Presence of felids | ^124^ |
|  |  | ＜2016 | Farm | ELISA^o^ | 201 | 12 | 6.0% | breeding style | ^140^ |
|  | **LiaoNing** | 2011 | Slaughter house | IHA^c^,1:64 | 646 | 39 | 6.04% | Geographical origin | ^101^ |
|  |  | 2013-2014 | Farm | IHA^a^, 1: 64 | 673 | 61 | 9.06% | Age, Gender, Geographical origin, species, season, abortion history, Source of water, Presence of felids | ^124^ |
| **Yaks** | | | | | | | | | |
| **Southwest China** | **SiChuan** | 2012 | Grazing | IHA^c^, 1: 64 | 212 | 54 | 25.47% | — | ^141^ |
|  |  | 2013 | Grazing | IHA^c^, 1: 64 | 252 | 85 | 33.73% | — | ^141^ |
|  | **Tibet** | 2012 | Grazing | IHA^c^, 1: 64 | 434 | 84 | 19.35% | — | ^141^ |
|  |  | 2013 | Grazing | IHA^c^, 1: 64 | 230 | 62 | 26.96% | — | ^141^ |
| **Northwest China** | **GanSu** | 2013-2014 | Farms | MAT, 1:100 | 974 | 155 | 15.91% | Age, Gender, season, pregnancy | ^142^ |
|  | **QingHai** | 2007 | — | IHA^c^, 1: 64 | 635 | 51 | 8.03% | — | ^8^ |
|  |  | <2008 | Grazing | IAT^c^, 1: 64 | 946 | 112 | 11.84% | Age | ^143^ |
|  |  | <2008 | Veterinary station | IHA^m^, 1: 64 | 43 | 29 | 67.44% | — | ^37^ |
|  |  | 2008-2009 | — | IHA^c^, 1: 64 | 100 | 12 | 12.15% | — | ^144^ |
|  |  | ＜2009 | — | ELISA^o^ | 1293 | 54 | 4.18% | — | ^132^ |
|  |  | 2009-2010 | Veterinary hospital | IHA^a^, 1: 64 | 1603 | 133 | 8.30% | Gender, Geographical origin, age | ^145^ |
|  |  | 2010 | — | IHA^c^, 1: 64 | 25 | 3 | 12.00% | Age, breed | ^134^ |
|  |  | 2010 | Grazing | IHA^b^, 1: 64 | 650 | 228 | 35.08% | Geographical origin | ^146^ |
|  |  | 2010 | Farm | ELISA^c^ | 898 | 21 | 2.34% | — | ^147^ |
|  |  | ＜2011 | Grazing and farms | IHA^c^, 1: 64 | 237 | 13 | 5.49% | — | ^13^ |
|  |  | 2012 | Grazing | IHA^c^, 1: 64 | 259 | 58 | 22.39% | — | ^141^ |
|  |  | 2013 | Grazing | IHA^c^, 1: 64 | 251 | 67 | 26.69% | — | ^141^ |

**Table S7 Seroprevalence of *T. gondii* infection in humans from China (2000–2017)**

| **Area** | **Province** | **Year** | **Source** | **Method, Titer** | **No. tested** | **No. positive** | **Positive (%)** | **Risk** | **References** |
| --- | --- | --- | --- | --- | --- | --- | --- | --- | --- |
| **East China** | **AnHui** | 2008 | HIV positive | ELISA^e^ | 302 | 4 | 1.32% | — | ^148^ |
|  |  |  | HIV negative |  | 302 | 1 | 0.33% |  |  |
|  |  | 2009-2010 | Resident | ELISA^e^ | 1795 | 201 | 11.20% | Age, High level of education | ^149^ |
|  |  | 2012-2013 | Cancer patients | ELISA^e^ | 1014 | 85 | 8.38% | — | ^150^ |
|  |  | 2016 | Young students | MAT, 1: 20 | 84 | 1 | 1.19% | North of the Yangtze River Province, keep a dog, Gardening or agriculture | ^151^ |
|  | **FuJian** | 2016 | Young students | MAT, 1: 20 | 12 | 0 | 0 | North of the Yangtze River Province, keep a dog, Gardening or agriculture | ^151^ |
|  | **JiangSu** | 2003 | Veterinarian, Breeder, Blood donor, Slaughterer, Pregnant woman, Tumor patients | ELISA^e^ | 450 | 34 | 7.56% | Veterinarian, Blood donor, Tumor patients | ^152^ |
|  |  |  | Resident |  | 450 | 18 | 4.00% |  |  |
|  |  | ＜2005 | Veterinarian, Breeder, Blood donor, Slaughterer, Pregnant woman, Tumor patients, Resident | ELISA^e^ | 3978 | 286 | 7.19% | Age | ^153^ |
|  |  | ＜2008 | Infertile couples | ELISA^e^ | 178 | 40 | 22.47% | infertility | ^154^ |
|  |  | 2012-2013 | Pregnant Women | ELISA^e^ | 1081 | 43 | 3.98% | — | ^155^ |
|  |  | 2015 | Pregnant Women,  Cancer patients,  Animal product processor | ELISA^e^ | 400 | 60 | 15.00% | Low level of education, cancer patients | ^156^ |
|  |  | 2015 | Pregnant Women,  Cancer patients,  Animal product processor | ELISA^e^ | 300 | 49 | 16.33% | Gender, Age, Level of education, Professional, The cutting board is cooked and raw separated | ^157^ |
|  |  | 2016 | Young students | MAT, 1: 20 | 17 | 0 | 0 | — | ^151^ |
|  |  | <2015 | HIV/AIDS patients | ELISA^e^ | 259 | 25 | 9.65% | — | ^158^ |
|  |  |  | Intravenous drug users |  | 90 | 2 | 2.22% |  |  |
|  |  |  | Healthy controls |  | 85 | 4 | 4.71% |  |  |
|  |  | <2016 | Pregnant Women,  Cancer patients,  Animal breeders | ELISA^—^ | 404 | 38 | 9.41% | Age, raw food, Contact the pets | ^159^ |
|  | **JiangXi** | 2003 | Resident | ELISA^e^ | 1960 | 157 | 8.01% | [Profession](file:///D:\%E7%94%B5%E8%84%91%E8%BD%AF%E4%BB%B6\youdao\Dict\7.3.0.0817\resultui\dict\?keyword=profession), Low level of education, raw food, hygiene, Feeding pet | ^160^ |
|  |  | 2014 | Pregnant Women,  Cancer patients,  Animal breeders,  Veterinarian, Blood donor, Resident | ELISA^e^ | 2400 | 124 | 5.17% | Profession, Low level of education, raw food, hygiene, Feeding cat | ^161^ |
|  |  | 2016 | Young students | MAT, 1: 20 | 10 | 0 | 0 | — | ^151^ |
|  | **ShanDong** | 2012-2014 | Cancer patients | ELISA^al^ | 900 | 342 | 38.00% | Cancer, Consumption raw or undercooked meat, Exposure with soil | ^162^ |
|  |  |  | Controls |  | 900 | 177 | 19.67% |  |  |
|  |  | 2014-2015 | Liver disease patients | ELISA^al^ | 744 | 182 | 24.46% | Region, Disease | ^163^ |
|  |  |  | Healthy controls |  | 876 | 111 | 12.67% |  |  |
|  |  | 2014-2016 | Tuberculosis patients | ELISA^al^ | 312 | 44 | 14.10% | Cats at home, Presence of stray cats, consumption of raw/undercooked meat | ^164^ |
|  |  |  | Healthy controls |  | 312 | 40 | 12.80% |  |  |
|  |  | 2016 | Young students | MAT, 1: 20 | 162 | 2 | 1.23% | — | ^151^ |
|  | **ShangHai** | <2004 | Veterinarian, Animal breeders, Resident | ELISA^e^ | 289 | 19 | 6.57% | — | ^165^ |
|  |  | 2003-2004 | Resident, Animal product processor, Animal breeders, Tumor patients | ELISA^e^ | 4169 | 168 | 4.03% | Low level of education, Feeding pets, Health habits | ^166^ |
|  |  | 2008-2009 | Veterinarian, Animal product processor, Chef | ELISA^e^ | 365 | 10 | 2.74% | Profession, female, Drinking | ^167^ |
|  |  | 2016 | Young students | MAT, 1: 20 | 16 | 1 | 6.25% | — | ^151^ |
|  | **ZheJiang** | 2000-2001 | Resident | ELISA^s^ | 1102 | 121 | 10.98% | Female, Profession | ^168^ |
|  |  | 2002 | Women, Animal product processor, Animal breeders, Resident | ELISA^—^ | 752 | 77 | 10.24% | Profession | ^169^ |
|  |  | 2002-2005 | Pregant women | ELISA^y^ | 5686 | 149 | 2.62% | Season, Profession, Abnormal pregnancy outcome | ^170^ |
|  |  | 2005 | Pregant women,  Abortion history | ELISA^—^ | 801 | 106 | 13.23% | — | ^171^ |
|  |  | 2010-2013 | Pregant women,  Healty control | ELISA^—^ | 1000 | 68 | 6.80% | Abnormal pregnancy outcome, Profession, Age, Low level of education, village  resident | ^172^ |
|  |  | 2012 | Pets breeder | ELISA^a^ | 528 | 16 | 3.03% | Age, Gender, Level of education, Eat raw meat | ^173^ |
|  |  | <2015 | Animal product processor, Animal breeders | ELISA^a^ | 810 | 43 | 5.31% | Age, Eat raw meat  Raw meat and cooked food mixed with chopping block, Exposure with soil | ^174^ |
|  |  | 2016 | Young students | MAT, 1: 20 | 44 | 1 | 2.27% | — | ^151^ |
| **North China** | **BeiJing** | 2016 | Young students | MAT, 1: 20 | 12 | 0 | 0 | — | ^151^ |
|  | **HeBei** | <2004 | Pregnant Women,  Cancer patients,  Blood donor, Resident | ELISA^e^ | 1300 | 86 | 6.62% | Feeding cat, Abnormal pregnancy history | ^175^ |
|  |  | 2006-2007 | Resident | ELISA^e^ | 3965 | 268 | 6.76% | Low level of education, Feeding pet, Health habits, unboiled water or milk, faggotry | ^176^ |
|  |  | 2007 | Hospital | ELISA^af^ | 637 | 40 | 6.28% | Age, Low level of education, Profession, Region, Health habits,  Raw meat and cooked food mixed with chopping block | ^177^ |
|  |  | <2012 | Undergraduate | ELISA^z^ | 478 | 11 | 2.30% | Age, Sleeping quality, eat raw meat, Contact pets | ^233^ |
|  |  | 2009 | Blood donor | ELISA^ae^ | 792 | 36 | 4.55% | Age | ^178^ |
|  |  | 2012 | Infertile couples | ELISA^e^ | 1474 | 118 | 8.01% | Infertility | ^179^ |
|  |  |  | Healthy control |  | 698 | 19 | 2.72% |  |  |
|  |  | 2014 | Blood donor | ELISA^a^^i^ | 832 | 35 | 4.21% | — | ^180^ |
|  |  | 2015-2016 | Blood donor | ELISA^ai^ | 1165 | 83 | 7.12% | Age, Profession, Level of education | ^181^ |
|  |  | 2016 | Young students | MAT, 1: 20 | 143 | 3 | 2.10% | — | ^151^ |
|  |  | 2016 | Blood donor | ELISA^ae^ | 1630 | 126 | 7.73% | Gender, Age, Profession, Level of education, Contact the pet, Eat Hot pot or Barbecue, Raw meat and cooked food mixed with chopping block, Bad hygiene | ^234^ |
|  | **Inner Mongolia** | 2008-2009 | Resident | ELISA^e^ | 350 | 39 | 11.14% | Level of education, Income, Feeding pets, Eat barbecue | ^182^ |
|  |  | 2016 | Young students | MAT, 1: 20 | 122 | 0 | 0 | — | ^151^ |
|  | **TianJin** | 2016 | Young students | MAT, 1: 20 | 45 | 1 | 2.22% | — | ^151^ |
|  | **ShanXi** | 2005-2007 | Eye diseases, | ELISA^ah^ | 160 | 20 | 12.50% | Eye diseases | ^183^ |
|  |  |  | Healthy controls |  | 160 | 9 | 5.63% |  |  |
|  |  | 2008 | Healthy controls | ELISA^ah^ | 858 | 35 | 4.08% | Age, Professional, Low level of education | ^184^ |
|  |  | <2012 | Resident | ELISA^e^ | 4018 | 510 | 12.69% | Life style, feeding pets, Eat raw meat or milk | ^185^ |
|  |  | 2012-2014 | Infertile patients | ELISA^e^ | 500 | 57 | 11.40% | Female, Contact the pets | ^186^ |
|  |  | 2016 | Young students | MAT, 1: 20 | 117 | 2 | 1.71% | — | ^151^ |
| **Central**  **China** | **HeNan** | 2002 | Resident | ELISA^—^ | 760 | 63 | 8. 29% | — | ^187^ |
|  |  | 2002-2004 | Resident | ELISA^—^ | 4660 | 216 | 4.64% | — | ^188^ |
|  |  | 2003-2005 | Infertile patients | ELISA^ac^ | 1411 | 127 | 9.00% | Female | ^189^ |
|  |  | 2008-2010 | AIDS patients | — | 1402 | 31 | 2.21% | — | ^190^ |
|  |  | 2014-2015 | Liver disease patients | ELISA^al^ | 398 | 51 | 12.81% | Region, Disease | ^163^ |
|  |  |  | Normal |  | 266 | 39 | 14.66% |  |  |
|  |  | 2016 | Young students | MAT, 1: 20 | 229 | 5 | 2.18% | — | ^151^ |
|  | **HuBei** | <2001 | Pets breeder | ELISA^m^ | 226 | 71 | 31.42% | Feeding cat or dog | ^191^ |
|  |  |  | Not breeding pets |  | 783 | 84 | 10.73% |  |  |
|  |  | 2003-2004 | Veterinarian, Animal breeders,  Butcher  Pregnant Women,  Blood donor | ELISA^e^ | 1798 | 117 | 6.51% | Contact animal or animal products | ^192^ |
|  |  |  | Resident |  | 1211 | 24 | 1.98% |  |  |
|  |  | 2002-2005 | Women | ELISA^x^ | 1018 | 51 | 5.01% | Miscarriage, Stillbirth | ^193^ |
|  |  | <2013 | Resident | ELISA^e^ | 3240 | 266 | 8.21% | Contact animal, Immunocompromised people, health habit | ^194^ |
|  |  | 2016 | Young students | MAT, 1: 20 | 18 | 0 | 0 | — | ^151^ |
|  | **HuNan** | 2001 | Worker from zoo,  Slaughterer,  Pet vendors | ELISA^e^ | 88 | 3 | 3.41% | Contact Feline | ^195^ |
|  |  | 1998-2009 | Children from hospital | ELISA^ag^ | 529 | 53 | 10.02% | Living village, Contact the pets | ^196^ |
|  |  | 2016 | Young students | MAT, 1: 20 | 22 | 1 | 4.54% | — | ^151^ |
| **South**  **China** | **GuangDong** | 2000-2001 | Healthy person | IHA^c^, 1: 64 | 1010 | 102 | 10.01% | profession | ^197^ |
|  |  | <2003 | Women, Slaughterer | ELISA^u^ | 380 | 22 | 5.79% | Female, working years | ^198^ |
|  |  | 2004 | Animal product processor, Animal breeders | ELISA^—^ | 232 | 21 | 9.05% | working years | ^199^ |
|  |  | 2003-2004 | Resident | ELISA^w^ | 504 | 28 | 5.56% | Slaughterer, Feeding cat and dogs | ^200^ |
|  |  | 2004 | Resident | ELISA^—^ | 2526 | 206 | 8.16% | Profession, Age | ^201^ |
|  |  | 2016 | Young students | MAT, 1: 20 | 1 | 0 | 0 | — | ^151^ |
|  | **GuangXi** | 2016 | Young students | MAT, 1: 20 | 26 | 1 | 3.85% | — | ^151^ |
|  | **HaiNan** | 2016 | Young students | MAT, 1: 20 | 9 | 1 | 11.11% | — | ^151^ |
|  | **ShenZhen** | <2005 | Blood donors | ELISA^y^ | 680 | 49 | 7.21% | — | ^202^ |
| **Southwest**  **China** | **ChongQing** | 2015 | Blood donors | ELISA^ak^ | 1001 | 78 | 7.79% | Profession, Level of education | ^203^ |
|  |  | 2016 | Young students | MAT, 1: 20 | 13 | 2 | 15.38% | North of the Yangtze River Province, keep a dog, Gardening or agriculture | ^151^ |
|  | **GuiZhou** | 2000-2006 | Pregnant women | ELISA^ad^ | 524 | 52 | 9.92% | Contact cat and dogs, Abnormal pregnancy | ^204^ |
|  |  | 2003-2004 | Resident | ELISA^z^ | 2936 | 496 | 16.89% | Slaughterer, Living village, female | ^205^ |
|  |  | 2003-2005 | Pregnant women, Animal breeders,  Immunocompromised people, Resident | ELISA^m^ | 4592 | 697 | 15.18% | — | ^206^ |
|  |  | 2016 | Young students | MAT, 1: 20 | 161 | 9 | 5.59% | North of the Yangtze River Province, keep a dog, Gardening or agriculture | ^151^ |
|  | **SiChuan** | 2016 | Young students | MAT, 1: 20 | 36 | 3 | 8.33% | — | ^151^ |
|  | **YunNan** | 2005-2007 | Psychopath | ELISA^a^ | 219 | 70 | 31.96% | Mental illness | ^207^ |
|  |  |  | Healthy control |  | 91 | 19 | 20.88% |  |  |
|  |  | 2010-2013 | Veterinarian, Animal product processor, Animal breeders,  Patients | ELISA^aj^ | 289 | 16 | 5.54% | Profession | ^208^ |
|  |  | <2014 | Resident | ELISA^e^ | 906 | 219 | 24.17% | Age, Profession, Contact free range swine | ^209^ |
|  |  | 2016 | Young students | MAT, 1: 20 | 18 | 1 | 5.56% | — | ^151^ |
| **Northwest**  **China** | **GanSu** | 2002 | Veterinarian, Animal product processor, Animal breeders,  Immunocompromised people | ELISA^e^ | 197 | 30 | 15.23% | Contacted cat and dog, Low immunity function, bad dietetic habit | ^210^ |
|  |  |  | Resident |  | 197 | 9 | 4.57% |  |  |
|  |  | 2002 | Women | ELISA^—^ | 498 | 27 | 5.42% | — | ^211^ |
|  |  | <2003 | Hospital | IHA^c^, 1: 64 | 308 | 21 | 6.82% | — | ^212^ |
|  |  | <2010 | Slaughter, Pregnant women, Animal breeders, Blood donor, Immunocompromised people | ELISA^e^ | 4040 | 343 | 8.49% | Profession, Level of education, Age | ^213^ |
|  |  | 2016 | Young students | MAT, 1: 20 | 72 | 0 | 0 | — | ^151^ |
|  | **QingHai** | 2000 | Resident | IHA^c^, 1: 64 | 455 | 7 | 1.54% | Muslim Chinese, cadres, age | ^214^ |
|  |  | 2010 | Pregnant women | ELISA^m^ | 51 | 4 | 7.84% | — | ^215^ |
|  |  | 2016 | Young students | MAT, 1: 20 | 13 | 0 | 0 | — | ^151^ |
|  | **ShaanXi** | 2006 | Blood donor | ELISA^ab^ | 368 | 30 | 8.15% | Profession | ^216^ |
|  |  | 2016 | Young students | MAT, 1: 20 | 23 | 2 | 8.69% | — | ^151^ |
|  | **XinJiang** | 2000 | Resident | IHA^c^, 1: 64 | 350 | 32 | 9.14% | — | ^217^ |
|  |  | 2003 | Resident | ELISA^e^ | 1387 | 83 | 5.98% | Muslim Chinese, Blood donor | ^218^ |
|  |  | <2011 | Resident | IHA^c^, 1: 64 | 336 | 18 | 5.36% | — | ^96^ |
|  |  | <2014 | Women | ELISA^m^ | 60 | 19 | 31.67% | — | ^219^ |
|  |  | 2016 | Young students | MAT, 1: 20 | 27 | 1 | 3.71% | — | ^151^ |
| **Northeast**  **China** | **HeiLongJiang** | 2014-2016 | Tuberculosis patients | ELISA^al^ | 303 | 45 | 14.9% | Cats at home, Presence of stray cats | ^164^ |
|  |  |  | Healthy control |  | 303 | 31 | 10.2% |  |  |
|  |  | 2016 | Young students | MAT, 1: 20 | 98 | 0 | 0 | — | ^151^ |
|  | **JiLin** | 2014-2016 | Tuberculosis patients | ELISA^al^ | 309 | 50 | 16.2% | Cats at home, Presence of stray cats | ^164^ |
|  |  |  | Healthy control |  | 309 | 33 | 10.7% |  |  |
|  |  | 2016 | Young students | MAT, 1: 20 | 77 | 1 | 1.29% | — | ^151^ |
|  | **LiaoNing** | 2003-2004 | Resident | ELISA^aa^ | 608 | 28 | 4.61% | Feeding pet | ^220^ |
|  |  | 2003-2004 | Slaughter, Pregnant women, Animal breeders, Hospital worker | ELISA^aa^ | 1216 | 56 | 4.61% | Feeding pet | ^221^ |
|  |  | <2004 | Cardiovascular disease patient | ELISA^x^ | 93 | 20 | 21.51% | Cardiovascular disease | ^222^ |
|  |  |  | Healthy control |  | 87 | 9 | 10.34% |  |  |
|  |  | 2016 | Young students | MAT, 1: 20 | 1901 | 27 | 1.42% | North of the Yangtze River, keep a dog, Gardening or agriculture | ^151^ |
|  | **NingXia** | 2010 | Resident | ELISA^a^ | 572 | 19 | 3.32% | Muslim Chinese | ^223^ |
|  |  | 2016 | Young students | MAT, 1: 20 | 17 | 0 | 0 | — | ^151^ |

**Method**

*IHA, indirect hemagglutination test; MAT, modified agglutination test; ELISA, enzymelinked immunosorbent assay;*

*^a^ ShenZhen Combined Biotech Co., Ltd., ShenZhen, China*

^b^ *Veterinary Research Institute, JiangSu Academy of Agricultural Sciences, NanJing, China*

*^c^ LanZhou Veterinary Institute, Academy of Agriculture and Science, China.*

*^d^ ShenZhen Green Shiyuan Biotechnology Co., Ltd, China*

*^e^* *ZhuHai Haitai Life Technology Company, China*

^f^ *LuoYang Lipusheng Information Co., Ltd. Production of IgG antigen test strips, China*

^g^ *Animal Husbandry of XinJiang Agricultural University, China*

^h^ *ShangHai fast spirit Biotechnology Co., China*

^i^ *ZhuHai S.E.Z. Haitai Biological Pharmaceuticals Co., Ltd., ZhuHai, China*

*^j^ ShenZhen Tongnuo biological technology co., Ltd., ShenZhen, China*

*^k^ ChongQing animal inspection station, China*

*^l^ Toxo Test-MT, Eiken Co., Ltd., Tokyo, Japan*

*^m^ in house*

*^n^ ShangHai Institute of Parasitic Diseases, China.
^o^ Japan National Soundtrack Animal Disease Research Control Center, Japan*

*^p^ IDEXX Laboratories, Inc., Westbrook, ME, USA*

*^q^ ShangHai Jiang Lai Biological Technology Co., Ltd, China*

*^r^ America Rapidbio Company, R&B Scientific, USA*

^s^ *ZheJiang Institute of Parasitic Diseases, ZheJiang, Province, China*

^t^ *State Key Laboratory of Agricultural Microbiology, HuaZhong Agricultural University, Wuhan, Hubei Province, China*

***^u^*** *Beijing university of science and technology of the Chinese academy of sciences, China*

*^v^ Guangdong zhuhai haitai biological products co. LTD, China*

*^w^ The parasitology department of southern medical university, China*

*^x^ Institute of parasitic diseases, ZheJiang medical academy, China*

*^y^ Italian DIESSE biotechnology company, Italy*

*^z^ Shenzhen green ham biotechnology co. LTD, China*

*^aa^ Jinmei bioengineering co. LTD, China*

*^ab^ HeNan huamei biological engineering co. LTD, China*

*^ac^ ShenZhen boca biotechnology co. LTD, China*

*^ad^ Beijing waugh and reproductive health biotechnology co. LTD, China*

*^ae^ Huamei bioengineering company, China*

*^af^ Beijing modern high biological products company, China*

*^ag^ Institute of parasitology of the institute, medical sciences of HuBei province, China*

*^ah^ ShenZhen krunda biological engineering co. LTD, China*

*^ai^ GuangZhou jianlun biotechnology co., LTD, China*

*^aj^ Germany wigrun research and development co., LTD, Germany*

*^ak^ Beijing bell bio-engineering co. LTD, China*

*^al^ Demeditec Diagnostics GmbH, Germany, China*

**Table S8 Summary the genotypes of *T. gondii* from food animals and geographical locations in China**

| **Distict** | **Province** | **Year for isolation** | **Host** | **Isolation/DNA, DNA source** | **Genotype** | **Refer** |
| --- | --- | --- | --- | --- | --- | --- |
| **East China** | **JiangXi** | 2010-2013 | Swine | 12DNAs, HLN | 12 ToxoDB #9 | ^224^ |
|  | **JiangSu** | <2009 | Swine | 1 isolates, — | 1 ToxoDB #10 | ^225^ |
|  | **AnHui** | <2013 | Chicken | 1 isolates, BR | 1 ToxoDB #225 | ^226^ |
| **Central China** | **HeNan** | 2006-2008 | Swine | 34 DNAs, (L, HLN, LU, AF, Spleen, ) | 34 ToxoDB #9 | ^227^ |
|  |  | 2010 | Swine | 13 DNAs, — | 6 ToxoDB #9,  7 ToxoDB #10 | ^228^ |
|  |  | 2015 | Cattle | 2 DNA, (BR, H, LU, L, S, K) | 2 ToxoDB #225 | ^24^ |
|  |  | 2015 | Swine | 5 DNA, HLN | 5 ToxoDB #9 | ^24^ |
|  |  | 2016 | Sheep | 2 isolates, tachyzoites | 2 ToxoDB #9 | ^229^ |
| **South China** | **HuNan** | <2009 | Swine | 1 isolates, — | 1 ToxoDB#10 | ^225^ |
| **Southwest China** | **GuangDong** | 2010-2013 | Swine | 1 DNAs , HLN | 1 ToxoDB #3 | ^224^ |
|  | **SiChuan** | 2010-2013 | Swine | 3 DNAs , HLN | 3 ToxoDB #9 | ^224^ |
|  | **ChongQing** | 2010-2013 | Swine | 1 DNAs , HLN | 1 ToxoDB #9 | ^224^ |
|  | **GuiZhou** | 2011-2012 | Swine | 5 isolates, (B, BR, H) | 5 ToxoDB #9 | ^230^ |
|  |  | <2013 | Swine | 4 isolates, (AF or BR) | 4 ToxoDB #9 | ^226^ |
|  | **YuNan** | 2011-2014 | Black goat | 8 DNAs, (HLN. Lu, L, LN) | 1 ToxoDB #9,  7 ToxoDB #10 | ^231^ |
| **Northwest China** | **GanSu** | <2009 | Swine | 1 isolates, , — | 1 ToxoDB#9 | ^225^ |
|  |  | 2015 | White Yak | 2 DNA, (H, L, Lu) | 2 ToxoDB #9 | ^142^ |
|  | **QingHai** | 2009 | Sheep | 1 isolates, (L, LG,S) | 1 ToxoDB#3 | ^225^ |
| **Northeast China** | **LiaoNing** | 2013-2014 | Swine | 15 isolates, H | 13 ToxoDB #9,  2 ToxoDB #3 | ^102^ |

AF: Ascitic fluids; B: Blood; BR: Brain; H: Heart; HLN: hilar lymph nodes; L: Liver; Lu: Lung; LN: Lymph nodes; LG: lymph gland; K: Kidney; S: Spleen

**Table S9 The seroprevalence of *T. gondii* in all food animals from 31 provinces (from high to low, respectively)**

| **Province/city** | **seroprevalence** | **Province/city** | **seroprevalence** | **Province/city** | **seroprevalence** | **Province/city** | **seroprevalence** |
| --- | --- | --- | --- | --- | --- | --- | --- |
| **ChongQing** | 67.17% (9460/14084) | **AnHui** | 22.74% (626/2753) | **Tibet** | 17.03% (283/1662) | **LiaoNing** | 9.46% (795/8404) |
| **SiChuan** | 32.88% (317/964) | **HeNan** | 22.04% (3272/14844) | **XinJiang** | 16.63% (1411/8487) | **TaiWan** | 9.11% (36/395) |
| **Guizhou** | 31.65% (3130/9889) | **HeBei** | 21.16% (1880/8884) | **JiLin** | 15.58% (583/3742) | **ShangHai** | 8.26% (100/1211) |
| **ZheJiang** | 29.63% (5128/17304) | **ShanDong** | 21.08% (598/2837) | **GuangXi** | 15.24% (159/1043) | **NingXia** | 7.40% (67/906) |
| **JiangSu** | 27.86% (1321/4742) | **FuJian** | 20.99% (475/2263) | **BeiJing** | 12.51% (139/1111) | **HeiLongJiang** | 4.91% (174/3541) |
| **HuBei** | 25.32% (1936/7645) | **JiangXi** | 20.14% (346/1718) | **GanSu** | 12.18% (829/6808) | **ShanXi** | 3.85% (1/26) |
| **YunNan** | 24.70% (1746/7070) | **ShaanXi** | 19.93% (205/1083) | **Inner Mongolia** | 12.09% (208/1721) | **TianJin** | — (0/29) |
| **HuNan** | 24.08% (848/3521) | **GuangDong** | 17.47% (489/2799) | **QingHai** | 11.56% (2766/23931) |  |  |

**References**

**References**

1. Dubey JP. Toxoplasmosis of animals and humans. 2nd Edn. Boca Raton, FL: CRC Press; Taylor & Francis Group 2010.
2. Liu J, Cai JZ, Yu JH, Xia ZF, Lin DG, Liu Q. Initial investigation of the serological epidemiology of *Toxoplasma gondii* in several animals. *Proc Nin Confe Chin Soc Vet Parasitol* 2006 (in Chinese).
3. Yang N, Hao P, Liu GZ, Zhao XL, Wang H, Liu Q. The first reported of serum antibody test of *Neospora caninum* and *Toxoplasma gondii* about sheep and goats in the area of Inner Mongolia. *Proc Elev Confe Chin Soc Vet Parasitol* 2011 (in Chinese).
4. Zou F, Yu X, Yang Y, Hu S, Chang H, Yang JF, Duan G. Seroprevalence and risk factors of *Toxoplasma gondii* infection in buffaloes, sheep and goats in Yunnan Province, Southwestern China. *Iran J parasitol* 2015; **10**(4): 648-651.
5. Lei CH, Bian SS, Gao D, Bao ZZ, Cai YQ. Serological survey of toxoplasmosis of sheep in Aletai region of Xinjiang. *Anim Husb Feed Sci* 2014; **35**(6): 99-100. (in Chinese).
6. Wang M, Ye Q, Zhang NZ, Zhang DL. Seroprevalence of *Toxoplasma gondii* infection in food-producing animals in Northwest China. [*Chin J Zoonoses*](http://c.wanfangdata.com.cn/Periodical-zgrsghbzz.aspx) 2016; **32**(7): 608-12 (in Chinese).
7. Han XM, Wu XH, Liu BR, Liu PY, Tang XY. Examination on zoonooes parasitic of livestock in Xining. [*Chin J Zoonoses*](http://c.wanfangdata.com.cn/Periodical-zgrsghbzz.aspx) 2001; **17**(4): 123-4 (in Chinese).
8. Cai JZ, Li CH. Serological investigation and epidemic situation of *Toxoplasma gondii* about livestock in Qinghai Province. *Proc First Confe Chin Soc Ani Husb Vet Pub Heal* 2008 (in Chinese).
9. Tie FP, Li C, Liu YL. Diagnosis of toxoplasmosis in sheep by ELISA. *Chin Herb Sci* 2011; **31**(3): 71-2 (in Chinese).
10. Li Y, Li ZK, Kang M, Li YJ, Liu LS. Serological survey of *Toxoplasmosa gondii* in Tibetan sheep in Huzhu County Qinghai Province. *Prog Vet Med* 2010; **31**(7): 119-21 (in Chinese).
11. Cai QG, Ma LQ. Establishment and serological investigation of ELISA method for the diagnosis of toxoplasmosis in sheep in Qinghai Province. *Chin J Vet Med* 2010; **46**(3): 39-40 (in Chinese).
12. Ma RL, Cai JS, Zhao QB, Hu GW, Li J, Li LF, Ma ZQ, Yuan YZ, Chen FJ, Pan XY. Serological investigation of toxoplasmosis in cattle and sheep in Delingha region, Qinghai Province. *Proc Elev Confe Chin Soc Vet Parasitol* 2011 (in Chinese).
13. Dong YS, Luo ZQ, Zhang GC, Liu XQ. Epidemiological survey of toxoplasmosis in cattle and sheep in Qinghai Province. *Chin J Zoonoses* 2011; **27**(4): 359 (in Chinese).
14. Ma LQ, Han XM, Cai QG, Zheng Y, Lu Y, Wang GP, Ye CY, Niu XY. Serum antibodies detection of *Toxoplasma gondii* in human and animals in some areas of Qinghai Province. *Chin J Vet Med* 2013; **49**(5): 49-50 (in Chinese).
15. Huang R, Li XP, Wang GH, Ma LQ. Detection of several pathogens about sheep in Gangcha, Qinghai Province. *Chin J Vet Med* 2015; **51**(4): 46-8 (in Chinese).
16. Gong HL. Serological detection of abortion disease in sheep in Qilian County of Qinghai Province. [*Chin Qinghai J Anim Vet Sci*](http://c.wanfangdata.com.cn/Periodical-qhxmsyzz.aspx) 2014; **44**(3): 12-3 (in Chinese).
17. Caiwang CL, Li XP, Wang GH, Ma LQ. Serological detection of abortion disease among sheep in Maqin County of Qinghai Province. [*Chin Qinghai J Anim Vet Sci*](http://c.wanfangdata.com.cn/Periodical-qhxmsyzz.aspx) 2015; **45**(4): 6-7 (in Chinese).
18. Li XP, Zeng B, Wang GH, Wang GP, Ma LQ. Serological detection of abortion disease in sheep in Tongde County of Qinghai Province. [*Chin Qinghai J Anim Vet Sci*](http://c.wanfangdata.com.cn/Periodical-qhxmsyzz.aspx) 2015; **45**(2): 8-9 (in Chinese).
19. Wang XY, Li XP, Cairang NJ,Wang GP, Wang GH, Ma LQ. Serological detection of abortion disease in Oura-type of Tibetan sheep in Henan County of Qinghai. [*Chin Qinghai J Anim Vet Sci*](http://c.wanfangdata.com.cn/Periodical-qhxmsyzz.aspx) 2015; **45**(4): 8-10 (in Chinese).
20. Li HQ. Inverstigation on the epidemic situation of chlamydia of sheep in Gonghe Country. *Shandong J Anim Sci Vet Med* 2016; **37**(10): 51-2 (in Chinese).
21. Lv CH, Xu XP, Sha WL, Sha WJ, Ha DLBK. Serological survey of toxoplasmosis in Altay region. *Xinjiang Farm Research of Science and Technology* 2006; (4): 41 (in Chinese).
22. Zhang YJ, Ma XH, Liu HM, Dou YX, Wang RJ, Zhao WS. Diagnosis and prevention of listeriosis in sheep. *Proc Confe Chin Soc Vet Sheep Prod Res in 2012*; 2012. p. 355-6 (in Chinese).
23. Mao KM, Lin ZH, Lin XM, Lin ZY, Chen Y, Wu D. Serological survey of chlamydia, toxoplasmosis and brucellosis in goats from Fuqing. [*J Anim Husb Vet Med Fujian*](http://c.wanfangdata.com.cn/Periodical-fjxmsy.aspx) 2013; (5): 21-2 (in Chinese).
24. Luo H, Li K, Zhang H, Gan P, Shahzad M, Wu XX, Lan YF, Wang JX. Seroprevalence of *Toxoplasma gondii* infection in zoo and domestic animals in Jiangxi Province, China. *Parasit (Paris, France)* 2017; **24**: 7.
25. Cui P, Fang SF, Wu ZY, Wu BQ. Epidemiological investigation of toxoplasmosis in farm animals of Hebei Province. *Chin Vet Sci* 2004; **34**(11): 33-4 (in Chinese).
26. Yang XY, Cui W, Yang LR, Wang R, Zhang XD, Liu ZL, Wang JG. Epidemiological suvery of parasitosis of domesticated goat in Engebei, Inner Mongolia. *Proc Nin Confe Chin Soc Vet Parasitol* 2006 (in Chinese).
27. Zhang D, Wang Z, Fang R, Nie H, Feng HH, Zhou YQ, Zhao JL. Use of protein AG in an enzyme-linked immunosorbent assay for serodiagnosis of *Toxoplasma gondii* infection in four species of animals. *Clin Vac Immunol: CVI* 2010; **17**(3): 485-6.
28. Li F, Wang SP, Wang CJ, He SC, Wu X, Liu GH. Seroprevalence of *Toxoplasma gondii* in goats in Hunan province, China. *Parasit (Paris, France)* 2016; **23**: 44.
29. Luo Y, Zhou BJ, Zhang H, Wang KG, Wen M, Chen ZT, Feng J, Jiang N, Zhang SX, Wang H. Epidemiological investigation of goat abortion in seven areas in Guizhou Province. *Anim Husb Vet Med* 2013; **45**(6): 62-65 (in Chinese).
30. Luo Y, Zhou BJ, Wang KG, Wen M, Cheng ZT, Zhang YX, Zhang H, Long CC, Jiang N. Comparison of IHA and ELISA in diagnosis of chlamydophila abortus and toxoplasmosis infections in goats. [*Prog Vet Med*](http://c.wanfangdata.com.cn/Periodical-dwyxjz.aspx) 2012; **33**(6): 49-51 (in Chinese).
31. Dong BY, Qian DX, Li GY, Wu YY, Wang XH, Chen B, Tan Q. Serological investigation of *Toxoplasma gondii* in Qiannan. [*Chin J Vet Med*](http://c.wanfangdata.com.cn/Periodical-zgsyzz.aspx) 2014; **50**(9): 33-4 (in Chinese).
32. Liu BS, Li YB, Wang QQ, Wang ZH, Guo XJ, Yang WR. Investigation of causes of caprine abortion in Honghe of Yunnan Province. *Chin Vet Sci* 2003; **33**(12): 31-5 (in Chinese).
33. Ouyang X, Xu C, Xue T, Yang YL, Lv R, Song HL, Sun SP. Serological survey of toxoplasmosis about goat in Yuxi City. [*Yunnan J Anim Sci Vet Med*](http://c.wanfangdata.com.cn/Periodical-ynxmsy.aspx) 2014; (3): 12-3 (in Chinese).
34. Wang QJ. An investigation of goat's toxoplasmosis in Chaidamu area. *Gansu Anim Vet Sci* 2002; **32**(3): 20 (in Chinese).
35. Wang GH, Xie CY, Ye CY, Han Y, Wang PG, Wang SL, Lu Y, Wang HZ, Cai QG, Li XP, Zhou JZ, Ma LQ. Investigation and prevention research on disease leading to goat abortion in Wulan County in Qinghai Province. [*Chin Qinghai J Anim Vet Sci*](http://c.wanfangdata.com.cn/Periodical-qhxmsyzz.aspx) 2013; **43**(3): 4-6 (in Chinese).
36. Li XH. Serological investigation of the causes of the abortion in cashmere goats in Haixi Prefecture of Qinghai Province. *Chin J Vet Med* 2007; **43**(8): 38 (in Chinese).
37. Chen CY. Epidemiological survey of livestock toxoplasmosis in Datong County. *Chin Qinghai J Anim Vet Sci* 2008; **38**(1): 23 (in Chinese).
38. Xu P, Li X, Guo L, Li B, Wang J, Yu D, Zhao Q, Liu XG. Seroprevalence of *Toxoplasma gondii* infection in Liaoning cashmere goat from northeastern China. *Parasit (Paris, France)* 2014; **21**: 22.
39. Zhao GH, Zhang MT, Lei LH, Shang CC, Cao DY, Tian TT, Li J, Xu JY, Yao YL, Chen DK, Zhu XQ. Seroprevalence of *Toxoplasma gondii* infection in dairy goats in Shaanxi Province, Northwestern China. *Parasit Vect* 2011; **4**: 47.
40. Liu X, Kan SH, Li RF, Zhu ZB, Liang XW, Lin Q. Serosurvey on *Toxoplasma gondii* infection in a dairy goat farm in Guanzhong region. [*Acta Agricul Boreali-occident Sin*](http://c.wanfangdata.com.cn/Periodical-xbnyxb.aspx) 2015; **24**(2): 16-9 (in Chinese).
41. Bai WS, Chen Y, Ba LT, Mai MT. Investigation of the livestock's toxoplasmosis by serological method in Akesu district. *Chin J Vet Parasitol* 2002; **10**(2): 29-30. (in Chinese)
42. Chen QL, Jier GL, Wang CM, Tureguli KLM, Ba YZH. Investigation report on *Toxoplasma gondii* infection in some mountain cashmere goats in Hejing County. *Contemp Anim Husb* 2014; (15): 18-9 (in Chinese).
43. Wang M. Serological determination on brucellosis, chlamydiosis and toxoplasmosis on Cashmere goat. [*Acta Ecol Anim Domast*](http://c.wanfangdata.com.cn/Periodical-jcst.aspx) 2011; **32**(6): 80-1 (in Chinese).
44. Xu P, Li X, Tang F, Liu YH, Kou X, Zhao ML, Li B, Guo L, Liu XG, Zhao Q. Seroprevalence and risk factors for *Toxoplasma gondii* in sheep and goats in Jinzhou, Northeastern China. *Trop Biomed* 2015; **32**(3): 563.
45. Ye KG, Fan F, Cui HP, Zhou QX. Serological investigation of *Toxoplasma gondii* infection in commercial slaughter in Wuxi City. *Livest Poul Indust* 2004; (5): 43 (in Chinese).
46. Xiang ZJ, Duan YL, Yue XJ, Chen PJ. Serological investigation of swine toxoplasmosis in partial area of Anhui Province. [*Hunan Agricul Sci*](http://c.wanfangdata.com.cn/Periodical-hunannykx.aspx) 2011; **2011**(17): 134-5 (in Chinese).
47. Wang H, Wang T, Luo Q, Huo XX, Wang L, Liu TT, Xu XC, Wang Y, Lu FL, Lun ZR, Yu L, Shen JL. Prevalence and genotypes of *Toxoplasma gondii* in pork from retail meat stores in Eastern China. *Int J Food Microbiol* 2012; **157**(3): 393-7.
48. Tang HP, Chen ZM, Xu ZS. Serological investigation and analysis of toxoplasmosis in Minnan area. *J Anim Husb Vet Med Fujian* 2005; **27**(6): 14 (in Chinese).
49. Zhang NG, Liu QL, Fan YC, Zhang ZL, Zhang XR. Serological survey of pig toxoplasmosis in Shanghang County. *J Anim Husb Vet Med Fujian* 2006; **28**(1): 34 (in Chinese).
50. Huang CQ, Lin YY, Dai AL, Li XH, Yang XY, Yuan ZG, Zhu XQ. Seroprevalence of *Toxoplasma gondii* infection in breeding sows in Western Fujian Province, China. *Trop Anim Health Product* 2010; **42**(1): 115-8.
51. Zhao ZX, Fan F, Zhou YH, Liu XB. Sera investigation on *Toxoplasma gondii* infections in pigs in Wuxi City. *Chin J Schistosom Cont* 2003; **15**(6): 417 (in Chinese).
52. Jiang HH, Zhang WB, Zhao L, Zhou DH, Song HQ, Xu CM, Deng SZ, Zhu XQ. Seroprevalence of *Toxoplasma gondii* infection in pigs in Jiangxi Province, Southeastern China. *Foodborne Path Dis* 2014; **11**(5): 362-5.
53. Chen YJ, Wang Q, Jiang W, Liu YC, Jing ZY, Yan YN. Investigation on swine toxoplasmosis in Shanghai suburbs. *Chin J Vet Parasitol* 2010; **18**(5): 54-8 (in Chinese).
54. Tsai YJ, Chung WC, Fei AC, Kaphle K, Peng S, Wu YL. Seroprevalence of *Toxoplasma gondii* in pigs from slaughterhouses in Taiwan. *J Parasitol* 2007; **93**(6): 1540-1.
55. Chen SF. Epidemiology and prevention and control measures of toxoplasmosis in the area of Ningbo City. [*Zhejiang J Anim Sci Vet Med*](http://c.wanfangdata.com.cn/Periodical-zjxmsy.aspx) 2014; **39**(5): 63-6 (in Chinese).
56. Yu HJ, Zhang Z, Liu Z, Qu DF, Zhang DF, Zhang HL, Zhou QJ, Du AF. Seroprevalence of *Toxoplasma gondii* infection in pigs, in Zhejiang Province, China. *J Parasitol* 2011; **97**(4): 748-9.
57. Huang LQ, Li H, He SG, Mao HR. Epidemiological investigation of toxoplasmosis in dogs, cats and swine in Zhejiang Province. *Chin J Vet Med* 2011; **47**(6): 39-41 (in Chinese).
58. Mu GL. Diagnosis and epidemiological investigation of swine toxoplasmosis in Yanqing County. *Chinese Acad Agricult Sci*; 2009 (in Chinese).
59. Yuan WY, Ma K, Yang HL. Surveys of *Toxoplasma gondii* infections in animals in Hebei Province. *Chinese J Schistosom Cont* 2004; **16**(1): 72 (in Chinese).
60. Cui P, Fang SF, Zhu JB. The serologic detection of pig’s *Toxoplasma gondii* in Hebei Province. *Chin J Vet Parasitol* 2004; **12**(3): 10-1 (in Chinese).
61. Gao CF, Kang GY, Qin JH, Gu XL. Detection of serum antibodies in toxoplasmosis in pigs and cows in Hebei Province. *Chin J Anim Quar* 2005; **22**(9): 39 (in Chinese).
62. Tao Q, Wang Z, Feng H, Fang HH, Fang R, Nie H, Hu M, Zhou YQ. Seroprevalence and risk ractors for *Toxoplasma gondii* infection on pig farms in central China. *J Parasitol* 2011; **97**(2): 262-4.

## Wang CM, He HX, Qin JH, Yao SX, Wang LR, Liu LY, Niu CF, Gao MC. Epidemiological investigation on *Toxoplasma gondii* infection in swine and sheep in the suburb of Xinxiang, Henan Province. *Chin J Parasitol Parasit Dis* 2005; 23(1): 31 (in Chinese).

## Shi DM, Guo YG, Hangpu HP, Fan YL. Clinical detection of pig serum samples infected with *Toxoplasma gondii.* *J Henan Agri Sci* 2006; 2006(4): 103-4 (in Chinese).

1. Wang TQ, Dong FM, Pan YQ, Zhang L, Fang JP. Serological survey and analysis of swine toxoplasmosis in Luoyang area. *Hubei J Anim Vet Sci* 2006; (9): 29-30 (in Chinese).

## Wang HY, Ren QE, Jan FC, Zhou Y, Ning CS, Zhang LX. Investigation on the swine *Toxoplasma gondii* infection in some districts of Henan Province and the inoculation experiment in mice infected with isolates from pigs. *Chin J Zoonoses* 2008; 24(10): 930-2 (in Chinese).

1. Jang SM, Cheng YP, Wang TQ. Serological survey and study of pig toxoplasmosis in Yichuan county. *J Anim Sci Vet Med* 2009; **28**(1): 35-6 (in Chinese).
2. Jia JY, Gao SZ, Shi WG, Zhang JJ. Investigation of sheep infectious and parasitic diseases in large-scale farms in Gansu province. *China Anim Health Inspect*. 2009, **26**(2): 44-46 (in Chinese).
3. Sun HY. Serological investigation of swine toxoplasmosis in Xinyang, Henan Province. *Prog Vet Med* 2016; **37**(9): 123-5 (in Chinese).
4. Du F, Zhang Q, Yu Q, Hu M, Zhou YQ, Zhao JL. Soil contamination of *Toxoplasma gondii* oocysts in pig farms in central China. [*Vet Parasitol*](https://www.ncbi.nlm.nih.gov/pubmed/?term=Development+of+reverse+transcription+loop-mediated+isothermal+amplification+(RT-LAMP)+as+a+diagnostic+tool+of+Toxoplasma+gondii+in+pork) 2012; **187**(1-2): 53-6.
5. Xu Y, Li RC, Liu GH, Cong W, Zhang XX, Yu XL, Zhu XQ. Seroprevalence of *Toxoplasma gondii* infection in sows in Hunan province, China. *Sci World J* 2014; **2014**: 347908.
6. Lao GY, Liu C, Xiao JH, Liao GY, Liu C, Xiao JH, Chen MH, Li YY, Chen MF. Serological survey of swine toxoplasmosis in Yongzhou, Hunan Province. *Chin J Anim Hus Vet Med* 2016; (7): 22-3 (in Chinese).
7. Xian QZ, Cai MF, Wang SM, Gu WJ. The serological investigation of swine toxoplasmosis. *Chin Anim Hus Vet Med* 2006; **33**(11): 103-4 (in Chinese).
8. Zhou DH, Liang R, Yin CC, Zhao FR, Yuan ZG, Lin RQ, Song HQ, Zhu XQ. Seroprevalence of *Toxoplasma gondii* in pigs from southern China. *J parasitol* 2010; **96**(3): 673-4.
9. Xu B, Zhang XL, Dong CX, Feng C, Su B, He Q, Zeng Z. Epidemiological investigation of swine toxoplasmamosis in Chongqing. *Prog Vet Med* 2013; **34**(3): 37-40 (in Chinese).
10. Tan QJ, li Y, Nie k, Zeng Z, Yang ZL. Epidemiological survey of *Toxoplasma gondii* in Chongqing. *Prog in Vet Med* 2008; **29**(7): 109-10 (in Chinese).
11. Wu D, Lv R, Sun X, Shu FF, Zhou ZY, Nie K, Duan G, Zou FC. Seroprevalence of *Toxoplasma gondii* antibodies from slaughter pigs in Chongqing, China. [*Trop Anim Health Prod*](https://www.ncbi.nlm.nih.gov/pubmed/?term=Seroprevalence+of+Toxoplasma+gondii+antibodies+from+slaughter+pigs+in+Chongqing%2C+China) 2012; **44**(4): 685-7.
12. Yang XW, Zhao GW. Investigation and analysis of the infection of *Toxoplasma gondii* in different rearing periods in Chongqing area. *Chin J Vet Med* 2015; **51**(2): 44-5 (in Chinese).
13. Hong NN, Qian DX, Zhang DX, Hua Y, Li T, Ran LZ, Tian HR, Liu X, Yu TD. Serological survey of porcine toxoplasmosiss infection in Guizhou Province. *Chin J Vet Parasitol* 2010; **18**(4): 68-70 (in Chinese).
14. Li HL, Dong L, Li Q, Zhang L, Chen J, Zou FC, Zhu XQ. Seroepidemiology of *Toxoplasma gondii* infection in Bai and Han ethnic groups in southwestern China. *Epidemiol Infect* 2015; **143**(4): 881-6.
15. Xiao FP, Yu B, Shi KZ, Yang YK, Yang MS, Xu HZ, Ren RQ. Serological survey and analysis of pig toxoplasmosis in Guiyang. *Anim Hus Vet Med* 2014; **46**(8) (in Chinese).
16. Wu YT, Xiao FP, Xie LL. Analysis of serological tests on swine toxoplasmosis in Guiyang. *Vet Ori* 2015; (10): 54-5 (in Chinese).
17. Meng BC, Wang JM, Zhou SH, Dong BY. Serological survey of swine toxoplasmosis in Qiannan prefecture. *Anim Hus Vet Med* 2016; **48**(6): 151-2 (in Chinese).
18. Li XL, Bu YM, Xie P, Hu SJ. Serological survey of swine toxoplasmosis in some pig farms in Sichuan Province. *Mod Livest Poul Breed Indust* 2010; (1): 96-7 (in Chinese).
19. Wu SM, Ciren D, Huang SY, Xu MJ, Ga G, Yan C, Mahmoud M, Zou FC, Zhu XQ. First report of *Toxoplasma gondii* prevalence in Tibetan pigs in Tibet, China. *Vect Bor Zoonot Dis (Larchmont, NY)* 2012; **12**(8): 654-6.
20. Ye YM, Wei DQ, Tu YF. Investigation of *Toxoplasma gondii* infected animals in Yunnan Province. *Chin J Parasitol Parasit Dis* 2002; **20**(4): 255.
21. Zou FC, Sun XT, Xie YJ, Li B, Zhao GH, Duan G, Zhu XQ. Seroprevalence of *Toxoplasma gondii* in pigs in southwestern China. *Parasitol Int* 2009; **58**(3): 306-7.
22. Deng ZH, Yang JF, Sun XT, Xie YJ, Zhao GH, Duan G, Zhou FC. Serological investigation of swine toxoplasmosis. *Chin Anim Hus Vet Med* 2010; **37**(8): 225-6 (in Chinese).
23. Liu M, Wan BY, Wei JH, Shu FF, Yang JF, Zhou FC. Serological investigation of toxoplasmosis in Yunnan Tibetan pigs. *Chin Livest Poul Breed* 2011; **07**(12): 111-2 (in Chinese).
24. He YC, Li S, Li XR, Qian ZB. Serological survey of animal toxoplasmosis in Zhangye city of Gansu Province. *Chin Anim Heal Inspect* 2016; **33**(3): 12-3 (in Chinese).
25. Ma LQ, Wang GP, Yan GF. Serum investigation on pig *Toxoplasma gondii* disease in Xining district of Qinghai Province. *J Dom Anim Ecol* 2003; **24**(4): 34-5 (in Chinese).
26. Fu YJ. Detection of the antibody levels against toxoplasmosis in swines, cattle and sheep in Qinghai Province. *Chin Vet Sci* 2003; **33**(9): 68-9 (in Chinese).
27. Yang YZ, Liu ZS, Li ZY, He QL. Serological investigation and prevention and cure of toxoplasmosis in Xining area. *Chin J Trad Vet Sci* 2004; (6): 10-2 (in Chinese).
28. Jian CR. Seroepidemiological investigation of *Toxoplasma gondii* infection in Chindu county of Qinghai Province. *Heilongjiang Anim Sci Vet Med* 2015; (16): 110-1 (in Chinese).
29. Wang J, Shan JJ, Jiang WS, Luo ZQ, Zheng CF, Gu X, Zhou HP. Serological investigation and analysis of toxoplasmosis in swine in Nongsanshi (Kashi), Xinjiang. *Chin Anim Heal Inspect* 2011; **28**(4): 62-3 (in Chinese).
30. Wang WS, Zhang JS, Chen W, Wang JW, Meng QL, Qiao J. Serological survey of toxoplasmosis infection among human and animals in Shihezi Region. *2nd Beijing For Trop Med Parasitol* 2010; 2010. p. 120-2 (in Chinese).
31. Lu GL, Wang W, Chen WD, Wang P, Shayilan KYZ, Cheng J, Xia J. Antibody monitor of *Toxoplasma gondii* in swine of some areas of Xinjiang. *Prog Vet Med* 2014; **35**(7): 125-7 (in Chinese).
32. Xu ZM, Wuerna M, Liu ML, Chen QL, Ba YCH. Serological detection of swine toxoplasmosis in Bazhou, Xinjiang. *Anim Hus Vet Med* 2016; **48**(9): 114-5 (in Chinese).
33. Chang QC, Zheng X, Qiu JH, Wang CR, Zhu XQ. Seroprevalence of *Toxoplasma gondii* infection in fattening pigs in Northeast China. *J Parasitol* 2013; **99**(3): 544-5.
34. Xu P, Cai YN, Leng X, Wang J, Ma W, Mu GD, Jiang J, Liu XY, Wang ZD, Zhao Q, Yang GL. Seroprevalence of *Toxoplasma gondii* infection in pigs in Jilin Province, Northeastern China. *Trop Biomed* 2015; **32**(1): 116-20.
35. Liu X, Liu C, Liu Y, Jin HT, Zhao YK, Chen J, Yang M, Quan L. Seroprevalence of *Toxoplasma gondii* infection in slaughtered pigs and cattle in Liaoning Province, northeastern China. *J Parasitol* 2012; **98**(2): 440-1.
36. Wang D, Liu Y, Jiang T, Zhang GX, Yuan GM, He JB, Su CL, Yang N. Seroprevalence and genotypes of *Toxoplasma gondii* isolated from pigs intended for human consumption in Liaoning province, northeastern China. *Parasit Vect* 2016; **9**: 248.
37. Shen B. Investigation on *Toxoplasma gondii* infection in chickens in some areas in China. *Nanjing Agri Col* 2010 (in Chinese).
38. Ding GE, Xu MB, Zhou YH, Fan F, Cui HP. Seroepidemiological survey of chickens infected with *Toxoplasma gondii* in Wuxi City. *Chin J Schistoso Cont* 2012; **24**(2): 243, 5.
39. Zhu J, Shen MH, Chen LE, Cao XY, Sun WM, Jin YC. Serological survey of *Toxoplasma gondii* infection in poultry in Songjiang District. *Shanghai J Anim Hus Vet Med* 2015; (6): 32-3 (in Chinese).
40. Tian PR, Cui P. Investigation on toxoplasmosis infection in chickens in Zhangjiakou city. *Anim Hus Feed Sci* 2010; **31**(9): 172-3 (in Chinese).
41. Cui P, Fang SF, Gu XL, Guo B, Sun XM. A survey of toxoplasmosis in chickens and rabbits in Bashang district, Shijiazhuang city. *Chin Anim Heal Inspect* 2010; **27**(5): 46-7 (in Chinese).
42. Zhao G, Shen B, Xie Q, Li XX, Yan RF, Song XK, Hassan IA, Li XR. Detection of *Toxoplasma gondii* in free-range chickens in China based on circulating antigens and antibodies. *Vet Parasitol* 2012; **185**(2-4): 72-7.
43. Jiang T, He HS. Epidemiological status and analysis of toxoplasmosis in Jingzhou city. *Anim Bred Feed* 2009; (12): 16-7 (in Chinese).
44. Luo H, Li K, Shahzad M, Zhang H, Lan YF, Xiong X. Seroprevalence of *Toxoplasma gondii* Infection in wild boars, wild rabbits, and wild chickens in Hubei Province, China. *Kor J Parasitol* 2017; **55**(1): 85-8.
45. Long X. Serological investigation of *Toxoplasma gondii* in Jingzhou city. *Yangtze Univ* 2013 (in Chinese).
46. Li JN. Analysis of *Toxoplasma gondii* infection and risk factors in Xinxiang area. *Xinxiang Med Col* 2015 (in Chinese).
47. Yan C, Yue CL, Yuan ZG, He Y, Yin CC, Lin RQ, Dubey JP, Zhu XQ. *Toxoplasma gondii* infection in domestic ducks, free-range and caged chickens in southern China. *Vet Parasitol* 2009; **165**(3-4): 337-40.
48. Liu RZ, Chen ZQ, Zhang CF, Zhong WC, Chen MX. Serological investigation of *Toxoplasma gondii* infection in some areas of Guangdong Province. *Poul Hus Dis Cont* 2013; (3): 7-9 (in Chinese).
49. Cong W, Huang SY, Zhou DH, Xu MJ, Wu SM, Yan C, Zhao Q, Song HQ, Zhu XQ. First report of *Toxoplasma gondii* infection in market-sold adult chickens, ducks and pigeons in northwest China. *Parasit Vect* 2012; **5**: 110.
50. Lei CH, Cai YQ, Bao ZZ, Bian SS, Gao D. Serological investigation on the infection of free-range chicken and sparrows toxoplasmosis in free-range chicken farm. *Heilongjiang Anim Sci Vet Med* 2015; (4): 71-2 (in Chinese).
51. Yang N, Mu MY, Li HK, He JB. Seroprevalence of *Toxoplasma gondii* infection in slaughtered chickens, ducks, and geese in Shenyang, northeastern China. *Parasit Vect* 2012; **5**: 237.
52. Wang DW, Han XH, Mu MY, Yuan GM, Zhang GX, He JB, Yang N, Li HK. Epidemiological investigation of toxoplasmosis in some animals in northeast China. *Heilongjiang Anim Sci Vet Med* 2014; (23): 129-31 (in Chinese).
53. Xu Y, Wang FY, Liu XY, Wei F, Liu Q. A modified agglutination test for diagnosing toxoplasmosis in chicken. *Chin J Vet Sci* 2014; **34**(11) (in Chinese).
54. Zhu JB, Yin JG, Xiao Y, Jiang N, Ankarlev J, Lindh J, Chen QJ. A seroepidemiological survey of *Toxoplasma gondii* infection in free-range and caged chickens in northeast China. *Vet Parasitol* 2008; **158**(4): 360-3.
55. Sun X, Wang Z, Li J, Wei F, Liu Q. Evaluation of an indirect ELISA using recombinant granule antigen GRA1, GRA7 and soluble antigens for serodiagnosis of *Toxoplasma gondii* infection in chickens. *Res Vet Sci* 2015; **100**: 161-4.
56. Yu JH, Xia ZF, Liu Q, Liu J, Ding J, Zhang W. Seroepidemiology of *Neospora caninum* and *Toxoplasma gondii* in cattle and water buffaloes (*Bubalus bubalis*) in the People's Republic of China. *Vet Parasitol* 2007; **143**(1): 79-85.
57. Yuan GM, Mu MY, Zhang GX, Yang N, Li HK. Investigation on *Toxoplasma gondii* infection in dairy cattle and beef cattle in Shandong Province. *Proceed Eleventh Conf Chin Soc Vet Parasitol*; 2013; 2013.
58. Sun WW, Meng QF, Cong W, Shan XF, Wang CF, Qian AD. Herd-level prevalence and associated risk factors for *Toxoplasma gondii*, *Neospora caninum*, *Chlamydia* abortus and bovine viral diarrhoea virus in commercial dairy and beef cattle in eastern, northern and northeastern China. *Parasitol Res* 2015; **114**(11): 4211-8.
59. Cao WB, Liu T, Zhang J, Wang M, Ji XY, Zhang Y, Zhao YL, Chang LY, Qian JH. Epidemiological survey of serum *Toxoplasma gondii* in some areas of Hebei Province. *Proceed Thirteenth Conf Chin Soc Vet Parasitol*; 2015; 2015.
60. Zhou XX, Zhou H, Ning XD, Li J, Jian FC, Zhang LX, Zhao QY, Ning CS. Seroprevalence of *Toxoplasma gondii* in cattle, sheep and goats in partial area of China. [*Chin Herb Sci*](http://c.wanfangdata.com.cn/Periodical-zgcsdw.aspx) 2014; (5): 43-6 *(in Chinese)*.
61. Wang J, Shi DM, Cheng HC, Huo J. Serological survey of toxoplasmosis of cattle in Zhengzhou City. [*Chin J Vet Med*](http://c.wanfangdata.com.cn/Periodical-zgsyzz.aspx) 2013; **49**(9): 39-40 (in Chinese).
62. Xu MJ, Liu QY, Fu JH, Nisbet AJ, Shi DS, He XH, Pan Y, Zhou DH, Song HQ, Zhu XQ. Seroprevalence of *Toxoplasma gondii* and *Neospora caninum* infection in dairy cows in subtropical southern China. *Parasitol* 2012; **139**(11): 1425-8.
63. Zhou DH, Zhao FR, Lu P, Xia HY, Xu MJ, Yuan LG, Yan C, Huang SY, Li SJ, Zhu XQ. Seroprevalence of *Toxoplasma gondii* infection in dairy cattle in southern China. *Parasit Vect* 2012; **5**: 48.
64. Li K, Han ZQ, Gao JF, Liu MY, Zhang D, Li JK. Seroprevalence of *Toxoplasma gondii* infection in cattle in some countries of Tibet, China. [*Chin Dai Cattl*](http://c.wanfangdata.com.cn/Periodical-zgnn.aspx) 2014; (18): 24-6 *(in Chinese)*.
65. Tan QD, Yang XY, Yin MY, Hu LY, Qian SY, Wang JL, Zhou DH, Zhu XQ. Seroprevalence and correlates of *Toxoplasma gondii* infection in dairy cattle in northwest China. *Acta Parasitol* 2015; **60**(4): 618-21.
66. Lu Y, Ma LQ. Serodiagnosis of toxoplasmosis and dovelopment of ELISA Kit. [*Chin J Anim Heal In*](http://c.wanfangdata.com.cn/Periodical-zgdwjy.aspx)*spect* 2009; **26**(10): 40-1 (in Chinese)*.*
67. Kang M, Li Y, Shi WH, Ren XR, Zhang CX, Zhang LJ. Serological investigation of toxoplasmosis in Hualong county, Qinghai Province. [*Anim Hus Vet Med*](http://c.wanfangdata.com.cn/Periodical-xmysy.aspx) 2013; **45**(10): 128 (in Chinese).
68. Zhao QB, Hu GW, Li J, Li LF, Ma ZQ, Yuan YZ, Chen FJ, Pan XY. Serological investigation of bovine toxoplasmosis in Delingha area of Qinghai Province. *Anim Hus Vet Med* 2011; **43**(4): 103-4 (in Chinese).
69. Chen CJ, Fan XL, Tan ML, Liu Q. Serological survey and analysis on brucella, chlamydia,and toxoplasmosis on scale dairy farming in Huangyuan county of Qinghai. [*Chin Qinghai J AnimVet Sci*](http://c.wanfangdata.com.cn/Periodical-qhxmsyzz.aspx) 2017; **47**(1): 33-5 (in Chinese).
70. Lv CH. The serology investigation of toxoplasmosis, brucellosis, chlamydiosis in cattle and sheep from Aletai area. *Xinjiang Agri Univ* 2006 (in Chinese).
71. Jiang YH, Wang ZR, Zhang yy, Ye Q, Fu XW. Serological investigation of *Toxoplasma gondii* in parts of Northern Xinjiang. *Chin J Vet Med* 2015; **51**(9): 56-7(in Chinese).
72. Qiu JH, Wang CR, Zhang X, Sheng ZH, Chang QC, Zhao Q, Wu SM, Zou FC, Zhu XQ. Seroprevalence of *Toxoplasma gondii* in beef cattle and dairy cattle in northeast China. *Foodborne Patho Dis* 2012; **9**(7): 579-82.
73. Ge W, Sun HC, Wang ZD, Xu P, Wang W, Mu GD, Wei F, Liu Q. Prevalence and genotype of *Toxoplasma gondii* infection in cattle from Jilin Province, northeastern China. *Vect Bor Zoonot Dis* 2014; **14**(6): 399-402.
74. Zhao P, Zhang SF, Jia LJ, Yu LZ, Li NL, Li JX. Serological investigation of bovine toxoplasmosis in Changchun, Jilin Province. [*Anim Hus Vet Med*](http://c.wanfangdata.com.cn/Periodical-xmysy.aspx) 2016; **48**(5): 146-7(in Chinese)*.*
75. Li K, Gao J, Shahzad M, Han ZQ, Nabi F, Liu MY, Zhang D, Li JK. Seroprevalence of *Toxoplasma gondii* infection in yaks (*Bos grunniens*) on the Qinghai-Tibetan Plateau of China. *Vet Parasitol* 2014; **205**(1-2): 354-6.
76. Qin SY, Zhou DH, Cong W, Zhang XX, Lou ZL, Yin MY, Tian QD, Zhu XQ. Seroprevalence, risk factors and genetic characterization of *Toxoplasma gondii* in free-range white yaks (*Bos grunniens*) in China. *Vet Parasitol* 2015; **211**(3-4): 300-2.
77. Liu J, Cai JZ, Zhang W, Liu Q, Chen D, Han JP, Liu QR. Seroepidemiology of *Neospora caninum* and *Toxoplasma gondii* infection in yaks (*Bos grunniens*) in Qinghai, China. *Vet Parasitol* 2008; **152**(3-4): 330-2.
78. Li Y, Li ZK, J H, Li YJ, Hou K. Serological survey of yak toxoplasmosis in Huzhu county, Qinghai Province. [*Chin J Anim Heal In*](http://c.wanfangdata.com.cn/Periodical-zgdwjy.aspx)*spect* 2010; 27(in Chinese).
79. Wang M, Wang YH, Ye Q, Meng P, Yin H, Zhang DL. Serological survey of *Toxoplasma gondii* in Tibetan mastiffs (*Canis lupus familiari*s) and yaks (*Bos grunniens*) in Qinghai, China. *Parasit Vect* 2012; **5**: 35.
80. Liu Q, Cai J, Zhao Q, Shang LM, Ma RL, Wang XL, Li J, Hu GW, Jin HT, Gao HW. Seroprevalence of *Toxoplasma gondii* infection in yaks (*Bos grunniens*) in northwestern China. *Trop Anim Heal Produc* 2011; **43**(4): 741-3.
81. Lu Y, Wang GP, Cai QG, Ye CY, Niu XY, MA LQ. Serological investigation of yak toxoplasmosis in cattle farm of Datong, Qinghai Province. [*Chin J Anim Heal In*](http://c.wanfangdata.com.cn/Periodical-zgdwjy.aspx)*spect* 2012; **29**(3): 40-1(in Chinese)*.*
82. Tian LG, Cheng GJ, Chen JX, Guo J, Tong XM, Cai YC, Liu Q, Zhou XN. Survey on co-infection with toxoplasmosis and HIV among people in China. *Chin J Schistoso Cont* 2010; **22**(4): 368-70(in Chinese).

## Liu DH, Wang TP, Guo JD, Cao ZG, Yin XM, Zhang YS, Wang FF, Liu XM. The serological survey of *Toxoplasma gondii* infection in Anhui Province. *J Trop Dis Parasitol* 2010; 08(4): 201-3(in Chinese)*.*

1. Wang L, He LY, Meng DD, Chen ZW, Wen H, Fang GS, Luo QL, Huang KQ, Shen JL. Seroprevalence and genetic characterization of *Toxoplasma gondii* in cancer patients in Anhui Province, Eastern China. *Parasit Vect* 2015; **8**(1): 162.
2. Yang N, Wang D, Xing M, Li CH, Li J, Wu AH, Sang XY, Feng Y, Jiang N, Chen QJ. Seroepidemiology and risk factors of *Toxoplasma gondii* infection among the newly enrolled undergraduates and postgraduate students in China. *Front Micro* 2017; **8**: 2092.
3. Zhang LX, Huang WG, Yuan JF. Study on *Toxoplasma gondii* infections in Wuxi. *Chin J Schistoso Cont* 2003; **15**(6): 466-7(in Chinese).
4. Xu XZ, Sun FH, Cao HJ, Qian YX, Chen JY, Sun MX, Zhao LF, Xu ZG. Investigation and study of sero-epidemiology on *Toxoplasma gondii* infection in special population. *J Trop Dis Parasitol* 2005; **3**(3): 133-5 (in Chinese).

## Xu XW. Detection of serum antibodies of *Toxoplasma gondii* in different populations of Suzhou city. *Chin J Schistoso Cont* 2008; 20(6): 487 (in Chinese).

1. Xia L, Wang YX. Investigation of *Toxoplasma gondii* infection in pregnant women in Wuxi. *J Trop Dis Parasitol* 2013; **11**(3): 169-70 (in Chinese).
2. Guo YL, Zou YG, Xv DC, Xu WW, Xie YQ, Dai Y, Shen MX. Analyais of *Toxoplasma gondii* infection and related factors among special population in Changzhou city, Jiangsu Province. *Chin J Schistoso Cont* 2016; **28**(6): 669-73 (in Chinese).
3. Guo YL, Xie YQ, Zou YG, Xu DC, Xu WW, Dai Y, Shen MX. Infection status of *Toxoplasma gondii* and its related knowledge and behavior among special population in Changzhou city. *Chin J Schistosom Cont* 2017; **29**(4): 498-501 (in Chinese).
4. Shen G, Wang X, Hui S, Gao Y. Seroprevalence of *Toxoplasma gondii* Infection among HIV/AIDS patients in Eastern China. *Kor J Parasitol* 2016; **54**(1): 93-6.
5. Gao DL, Meng XJ, Zhang X, Qian YH, Lu B. Survey on *Toxoplasma gondii* infection among key populations in Wuxi city. *Chin J Schistoso Cont* 2017; **29**(3): 352-4 (in Chinese).
6. Chen HY, Xiao HM, Zeng XJ, Xiong CH, Ge J, Hu GH. Seroepidemiologic investigation on the infection of *Toxoplasma gondii* among population in Nanchang area. [*Chin J Parasit Dis Cont*](http://www.sogou.com/link?url=WaeIF24cBDvLg2SclOcgDUC4_7MlSOJvr-B_mK8HAJ2X4UtOfkKM83p9IhmiUppHSv8vTv4dkmyZSMkEd_9W0vxpTtfLoYiL) 2005; **18**(6): 435-7 (in Chinese).
7. Peng GH, Hu zh, GE J, Hang CQ, Fu RL, Feng XW. Investigation on human *Toxoplasma gondii* infections in Nanchang city in 2014. *Chin J Schistoso Cont* 2016; **28**(5): 545-9 (in Chinese).
8. Cong W, Liu GH, Meng QF, Dong W, Qin SY, Zhang FK, Zhang XY, Wang XY, Qian AD, Zhu XQ. *Toxoplasma gondii* infection in cancer patients: prevalence, risk factors, genotypes and association with clinical diagnosis. *Cancer Lett* 2015; **359**(2): 307-13.
9. Tian AL, Li GX, Elsheikha HM, Zhang XY, Dong W, Yang PX, Luo YY, Li LH, Cong W, Zhu XQ. Seroepidemiology of *Toxoplasma gondii* infection in patients with liver disease in eastern China. *Epidemiol Infect* 2017; **145**(11): 2296-302.
10. Zhao YJ, Zhao YH, Zhang XY, Sun XJ, Liu YQ, Hou YJ, Wu JQ, Jia H, Han YN, Dong W, Yan SM, Qian AD. First Report of *Toxoplasma gondii* infection in tuberculosis patients in China. *Vect Borne Zoonot Dis (Larchmont, NY)* 2017.
11. Wang WY, Liu YN, Shen HG, Zhao DX, Pan JQ. Investigation of *Toxoplasma gondii* infection in special occupations in Minhang district, Shanghai. *Chin J Schistoso Cont* 2004; **16**(5): 342(in Chinese).
12. Ma XB, Cai L, Zhang BX, Fu YH, Chen J, Cao L, Jiang XJ. A survey on *Toxoplasma gondii* infection of human in Shanghai. *Shanghai J* *Pre Med* 2006; **18**(10): 483-6 (in Chinese).
13. Chen Y, Li Y, Zhang W, Zhang JM, Tao LX, Song WM. Infection and risk assessment of *Toxoplasma gondii* in key occupational groups in Fengxian district, Shanghai. [*Chin J Parasit Dis Cont*](http://www.sogou.com/link?url=WaeIF24cBDvLg2SclOcgDUC4_7MlSOJvr-B_mK8HAJ2X4UtOfkKM83p9IhmiUppHSv8vTv4dkmyZSMkEd_9W0vxpTtfLoYiL) 2011; (10): 767-8 (in Chinese).
14. Zhan FC, Shao JQ, Han CX, Lu B, Zhan ZX. Epidemiological survey of *Toxoplasma gondii* infection in Changshan county. *Chin J Schistoso Cont* 2003; **15**(4): 308-9 (in Chinese).
15. Zhao LL, Qian XJ. Investigation on the infection of *Toxoplasma gondii* in key population of Xiangshan county*. Zhejiang Pract Med* 2007; **12**(6): 441-2 (in Chinese).
16. Zhou QX, Wu JH. Relationship between *Toxoplasma gondii* infection and adverse pregnancy outcome in pregnant women. *Chin J Public Health* 2007; **23**(1): 29 (in Chinese).
17. Zhou SJ. Analysis of 801 cases of *Toxoplasma gondii* infection in obstetrics. *Chin J Rurl Med Pha* 2007; **14**(3): 25 (in Chinese).
18. Ye Y, Zhang L, Huang GY, Li X. Investigation on current situation of *Toxoplasma gondii* infection in reproductive population. [*Chin J Woman Child Health Res*](http://c.wanfangdata.com.cn/Periodical-gwyx-fybj.aspx) 2015; (3): 462-4 (in Chinese).
19. Wang J, Xu WM, Wang H, Yang Y, Jin XY, Huang YM. A survey on *Toxoplasma gondii* infection of pet feeders in Hangzhou in 2012. *Chin J Health Lab Technol* 2013; (11): 2516-8 (in Chinese).
20. Wang J, Xu WM, Wang H, Tang Y, Yang Y, Huo LL. Analysis of *Toxoplasma gondii* infection characteristics among high risk population in Hangzhou. *Chin J Health Lab Technol* 2015; (13): 2179-81 (in Chinese).
21. Wang YX. Investigation on *Toxoplasma gondii* infection in specific population in Chengde area. *Hebei Med Univ*; 2004 (in Chinese).
22. Du M, Liu SJ, Zhang BZ, Li WH, Xu BH. Investigation on the status of *Toxoplasma gondii* in different population in Shijiazhuang city. *Parasit Infect Dis* 2008; **6**(2): 103-4 (in Chinese).
23. Xu ZL, Sun FE, Hu XM, Jin Y, Chen RL, Zhu FL, Liu YX. Serological survey of antibodies IgG and IgM to *Toxoplasma gondii* among population of Cangzhou city. *J Envir Health* 2009; **26**(2): 151-2 (in Chinese).
24. Song RH. Analysis of the Toxoplasma gondii antibodies in different donors. *Chin J Blood Transf* 2009; **22**(10): 835-6 (in Chinese).
25. Li XJ, Xu T, Song RH. Investigation on *Toxoplasma gondii* infection among patients with infertility in Chengde, Hebei Province. [*Int J Med Parasit Dis*](http://c.wanfangdata.com.cn/Periodical-gwyx-jscbfc.aspx) 2014; **41**(5): 276-8 (in Chinese).
26. Wang YR, Shi HL, Chen YK, Yang YX, Song RH. Serological survey of *Toxoplasma gondii* infection in healthy blood donation population. [*Med Recap*](http://c.wanfangdata.com.cn/Periodical-yxzs.aspx) 2014; **20**(21): 3992-3 (in Chinese).
27. Shen YY, Yang YX, Chen YK, Song XD. Investigation on *Toxoplasma gondii* infection in healthy voluntary blood donors in Baixiang, Hebei Province. *Chin J Pest Cont* 2017; (10): 1101-2 (in Chinese).
28. Yu JF, Shi ZB, Gao QR, An CY, Mu L. Investigation and analysis of *Toxoplasma gondii* in Hohhot. *J Inner Mongol Med Univ* 2011; **33**(3): 229-31 (in Chinese).
29. Zhang SP, Yue HP, Hou YY, Zhao JY, Zhang QH, Xu XW, Yang R, Hao YX, Zhao YP, Zhang J. Serological survey of ocular toxoplasmosis in Shanxi. [*Chin J Parasit Dis Cont*](http://www.sogou.com/link?url=WaeIF24cBDvLg2SclOcgDUC4_7MlSOJvr-B_mK8HAJ2X4UtOfkKM83p9IhmiUppHSv8vTv4dkmyZSMkEd_9W0vxpTtfLoYiL) 2011; **06**(1): 52-4 (in Chinese).
30. Yue HP, Hou YY, Rao HX, Hao YX, Guo JK, Zhang J, Chang HY, Chen GG, Xing XW. Investigation of *Toxoplasma gondii* infection in healthy people in Taiyuan. [*Chin J Parasit Dis Cont*](http://www.sogou.com/link?url=WaeIF24cBDvLg2SclOcgDUC4_7MlSOJvr-B_mK8HAJ2X4UtOfkKM83p9IhmiUppHSv8vTv4dkmyZSMkEd_9W0vxpTtfLoYiL) 2011; (4): 291-3 (in Chinese).
31. Liu Y, Study on the infection characteristics and related factors of *Toxoplasma gondii* in special and general population of Shanxi. *Shanxi Med Univ* 2006 (in Chinese).
32. Zhang HF, Zhang S, Yang XL, Wang ZB. Analysis of *Toxoplasma gondii* infection in infertile people. *Chin Rem Clin* 2015; (1): 77-8 (in Chinese).
33. Li H, Xu BL, Zhao XD, Lin XM, Huang Q, Deng Y, Wang H, Hao JL, Liang ZS. Analysis on current status of main human parasities disease in Zhoukou in 2002. *Prevent Med Trib* 2005; **11**(5): 528-30 (in Chinese).
34. Xu BL, Zhao XD, Su YP, Li H, He LJ, Lin XM, Yan QY, Huang Q, Yan XX, Liu H, Chen JS, Wang H, Deng Y, Lu DL, Li P, Li DF, Shang LY, Li Y. Survey on present epidmic status of main human parasites in Henan Province. *J Parasit Biol* 2005; **18**(6): 454-7 (in Chinese).
35. .Liu RF, Wang XC. Detection and analysis of IgM antibodies against *Toxoplasma gondii* in infertile patients. *J Henan Univ Sci Technol (Med Sci)* 2007; **25**(4): 279-80 (in Chinese).
36. Liu CL, Liu XH, Guan Q, Hou MJ, Fan SQ, Zhao QX, Sun Y. Clinical analysis of 31 cases of AIDS and *Toxoplasma gondii* encephalitis. *Chin J Pract Nerv Dis* 2013; **16**(3): 45-7 (in Chinese).
37. Chen CY. The study of individual having pet tend to *Toxoplasma gondii* infection. *Chin J Zoonoses* 2001; **17**(1): 76-7 (in Chinese).
38. Yu PH, Chen JS, Zhang HX, Zhang C, Wang CX, Mao CX, Xu MX. The serological survy of *Toxoplasma gondii* infection in Wuhan city. *Chin J Zoonoses* 2007; **23**(4): 393-4 (in Chinese).
39. Yin WP, Mao HL, Kong XL. Examining toxoplasmosis of 1018 women of child-bearing age. *J Public Health Prevent Med* 2005; **16**(6): 57 (in Chinese).
40. Zhu J, Wei RH, Yang SG, Zhu MS. Seroepidemiological survey on *Toxoplasma gondii* infection in Shiyan. *J Hubei Univ Med* 2013; (3): 199-200 (in Chinese).
41. Duan JH, Zhuang SF, Li ZX, Long HY, Zheng J. Survey of *Toxoplasma gondii* infection in special population of animal breeders and slaughter workers in Changsha city. *Pract Prevent Med* 2003; **10**(5): 774 (in Chinese).
42. Shi M, Wang LB, Mei SH, Zhang KR, Li LL, Ye MW. Investigation and analysis of *Toxoplasma gondii* infection in Xiangxi. *Practl J Card Cereb Pneu Vascul Dis* 2010; **18**(5): 586-7 (in Chinese).
43. Guo Y, Wang SQ, Jin LQ, Chen JQ. Application of ELISA and IHA on detecting the antibodies against *Toxoplasma gondii* in the sera of humans in the Hilly Land of Meizhou in East Guangdong Province. *Dis Surv* 2002; **17**(7): 247-9 (in Chinese).
44. Lu GP, Zhou YM, Zhou CH, Wu AL. Study on the infection and control of *Toxoplasma gondii* in major population of Jiangmen city. *J Trop Med* 2002; **2**(3): 309-11 (in Chinese).
45. Zhao LQ, Liu SJ. Serological surveillance of *Toxoplasma gondii* infection in Haizhu district in 2004. *J Trop Med* 2007; **7**(5): 495-6 (in Chinese).
46. Xie XQ, Deng YH, Xiao KG, Huang HQ, Huang JP, Lu JW, Gu XZ, Li CQ. Study on the *Toxoplasma gondii* infection and its control in Meixian. *J Trop Med* 2004; **4**(6): 731-2 (in Chinese).
47. Feng YJ, Liu XN, Ren WF, Pan ZM, Gao YP, Guo RT. Serological Analysis of toxoplasmosis in Guangzhou city in 2004. *J Trop Med* 2005; **5**(6): 830-1 (in Chinese).
48. Zeng JF, Li H, Cai H, Chen G. Seroepidemiological survey of *Toxoplasma gondii* in infection in blood donors in Shenzhen city. *Chin Trop Med* 2005; **5**(3): 599-600 (in Chinese).
49. Xu M, Mao W, He T, Zhang CH, Wei YY, Gao Z, Zhang Y, He M. Investigation and research on *Toxoplasma gondii* infection of volunteer blood donors in Chongqing, China. *Chin J Blood Transfusion* 2017; **30**(1): 58-60 (in Chinese).
50. Zheng YX. Analysis of *Toxoplasma gondi* infection in pregnant women: A report of 78 cases. *Guizhou Med J* 2007; **31**(1): 78 (in Chinese).
51. Chen ZY, Li AM, Lin GC, Wang XZ. Epidemiological investigation on the infection of *Toxoplasma gondii* from different populations in Guizhou Province. *J Zunyi Med Univ* 2005; **28**(4): 382-3 (in Chinese).
52. Wang SH, Chen ZY. Seroepidemiological investigation of *Toxoplasma gondii* infection in some population in Guizhou Province. *Guizhou Med J* 2007; **31**(8): 748-9 (in Chinese).
53. Li W, Shen LJ. Investigation of *Toxoplasma gondii* infection in psychopath from Dali. *J Dali Univ* 2008; **7**(8): 25-6 (in Chinese).
54. Wang YB, Yang XD, Yang GR, Li WG, Yang SB, Peng HY, Yu BB, Li ZQ, Jeffrey G, Fred U. Study on the prevalence of human toxoplasmosis in different ecological environment in Yunnan Province. *Pract Prevent Med* 2015; **22**(8): 918-20 (in Chinese).
55. Wu FW, Wang LB, Du ZW, Zhou HN, Jiang JY, Mao XH, Wang XZ. Serological survey of *Toxoplasma gondii* infection in some areas of Pu’er city. *Chin J Schistosom Cont* 2014; **26**(5): 583-5 (in Chinese).
56. Zhang Y, Li H. Epidemiological investigation on the toxoplasmosis among high risk group in Lanzhou district. *Chin J Parasit Dis Contl* 2005; **18**(6): 432 (in Chinese).
57. Zhang Y, Li H. Investigation on *Toxoplasma gondii* infection in pregnant women in Lanzhou. *Health Vocat Educat* 2005; **23**(23): 81-2 (in Chinese).
58. Zhang DL, Dou YX, Li XR, Zhao HY, Yang M. Serological survey of toxoplasmosis in Lanzhou population. *Prog Vet Med* 2003; **24**(6): 129 (in Chinese).
59. Li F, Feng Y, Chen SB, Ren DY, Yang CM, Yang JK. Survey of *Toxoplasma gondii* infection on special population in three cities of Gansu Province. *Chin J Schistosom Cont* 2010; **22**(3): 288-90 (in Chinese).
60. Cai HX, He DL, Wu XH, Han XM, Ning G. A Seroepidemiological survey on human toxoplasmosis in Hualong County, Qinghai Province. *J Trop Dis Parasitol* 2003; **1**(2): 92-3 (in Chinese).
61. Ma LQ, Han XM, Cai QG, Zheng Y, Lu Y, Wang GP, Ye CY, Niu XY. Serological detection of toxoplasmosis in human and animal in Qinghai Province. *Anim Husb Vet Med* 2013; **45**(2): 74-6 (in Chinese).
62. Ai CL, Hui QF, Sun XM, Xue YJ, Jing CX. Investigation and research on *Toxoplasma gondii* infection of blood donors in Yan'an city. *Shaanxi Med J* 2007; **36**(7): 884-6 (in Chinese).
63. Zhao HZ, Ren DK. Survey on toxoplasmosis of key population in Hami of Xinjiang in 2000. *Endem Dis Bullet* 2005; **20**(4): 36-7 (in Chinese).
64. Zhang S, Tong SX, Menghe BT, Yisilayin WSM, Niu XL, Si KDE, Li X, Ruziguli ZMH, Cheng FQ, Ma JP . Seroepidemiological study on toxoplasmosis in North slope of Tianshan Mountain in Sinkiang. *Endem Dis Bullet* 2008; **23**(2): 35-6 (in Chinese).
65. Ma YL, Abu LT. Investigation of the toxoplasmosis infection in people, dogs, cats in Kuqa County. *Xinjiang Agri Univ* 2014 (in Chinese).
66. Wang WZ, Wang XH, Nian F, Zhang XL, Chen SY. Study on the *Toxoplasma gondii* infection in the high-risk population in Jinzhou. *J Trop Med* 2006; 6(9): 1011-2 (in Chinese).
67. Wang WZ, Nian F, Liang W, Liu YH, Wang YQ, Wang XH, Chen SY, Cui CX. Study on the *Toxoplasma gondii* infection in the high-risk population in the West of Liaoning. *Mord Pre Med* 2007; **34**(9): 1745-6 (in Chinese).
68. Yu J, Li YJ, Liu T, Wang SH, Ma SQ. Detection of *Toxoplasma gondii* IgG, IgM and circulating antigens in patients with cardiovascular diseases in Dalian. *Pract Pre Med* 2004; **11**(6): 1196-7 (in Chinese).
69. Yang J, Song L, Zhang M, Song XJ, Ma XP, Ma LY, Li L. Survey on *Toxoplasma gondii* infection in rural residents and livestock in Hongsi Fort, Ningxia. *J Ningxia Med Univ* 2012; **34**(1): 46-8 (in Chinese).
70. Jiang HH, Huang SY, Zhou DH, Zhang XX, Su CL, Deng SZ, Zhu XQ. Genetic characterization of *Toxoplasma gondii* from pigs of different localities in China by PCR-RFLP. [*Parasit Vectors*](https://www.ncbi.nlm.nih.gov/pubmed/?term=Seroprevalence+and+genotype+of+Toxoplasma+gondii+in+pigs%2C+dogs+and+cats+from+Guizhou+province%2C+Southwest+China) 2013; 6:
71. Zhou P, Zhang H, Lin RQ, et al. Genetic characterization of *Toxoplasma gondii* isolates from China. *Parasitol Int* 2009; **58**(2): 193-5.
72. Wang L, Cheng HW, Huang KQ, Xu YH, Li YN, Du J. Li Y, Luo QL, Wei W, Jiang L, Shen JL. *Toxoplasma gondii* prevalence in food animals and rodents in different regions of China: isolation, genotyping and mouse pathogenicity. [*Parasit Vectors*](https://www.ncbi.nlm.nih.gov/pubmed/?term=Seroprevalence+and+genotype+of+Toxoplasma+gondii+in+pigs%2C+dogs+and+cats+from+Guizhou+province%2C+Southwest+China) 2013; 6: 273.
73. Wang H, Zhang L, Ren Q, Yu FC, Yang YR. Diagnosis of swine toxoplasmosis by PCR and genotyping of *Toxoplasma gondii* from pigs in Henan, Central China. [*BMC Vet Res*](https://www.ncbi.nlm.nih.gov/pubmed/?term=Diagnosis+of+Swine+Toxoplasmosis+by+PCR+and+Genotyping+of+Toxoplasma+gondii+from+pigs+in+Henan%2C+Central+China) 2017; **13**(1): 152.
74. Zhou P, Nie H, Zhang LX, . Genetic characterization of *Toxoplasma gondii* isolates from pigs in China. *J parasitol* 2010; **96**(5): 1027-9.
75. Yang YR, Feng YJ, Qiu XY, Wang YH, Lu YY, Liang HD, Zhu XQ, Zhang LX. Seroprevalence, isolation, genotyping, and pathogenicity of *Toxoplasma gondii* strains from sheep in China. *Front Microbiol* 2017; 8: 136.
76. Li YN, Nie XW, Peng QY, Mu XQ, Zhang M, Tian MY, Min SJ. Seroprevalence and genotype of *Toxoplasma gondii*, in pigs, dogs and cats from Guizhou province, Southwest China. [*Parasit Vectors*](https://www.ncbi.nlm.nih.gov/pubmed/?term=Seroprevalence+and+genotype+of+Toxoplasma+gondii+in+pigs%2C+dogs+and+cats+from+Guizhou+province%2C+Southwest+China) 2015, **8**(1):1-5.
77. Miao Q, Huang SY, Qin SY, Yu X, Yang Y, Yang JF, Zhu XQ, Zou FC. Genetic characterization of *Toxoplasma gondii* in Yunnan black goats (Capra hircus) in southwest China by PCR-RFLP. [*Parasit Vectors*](https://www.ncbi.nlm.nih.gov/pubmed/?term=Seroprevalence+and+genotype+of+Toxoplasma+gondii+in+pigs%2C+dogs+and+cats+from+Guizhou+province%2C+Southwest+China) 2015; 8: 57.
78. Feng Y, Lu Y, Wang Y, Liu J, Zhang L, Yang Y. *Toxoplasma gondii* and *Neospora caninum* in Free-Range chickens in Henan province of China. *Biomed Res Int* 2016; **2016**: 8290536.
79. Shen Y, Yuan WY, Wu H, Guo HT, Wei ZY, Ma Q. Survey on the infection situation of *Toxoplasma gondii* among college students in Baoding city. *Med Res Edu* 2012, **29**(1): 51-55 (in Chinese).
80. Wu Y，Song RH. Survey of *Toxoplasma gondii* infection characteristics and its risk factors among healthy blood donation population in Shijiazhuang City，Hebei Province. *Chin J Schisto Control* 2017, **29**(3): 377-379 (in Chinese).
